# Numerical Simulation of Fracking in Shale Rocks: Current State and Future Approaches

**DOI:** 10.1007/s11831-016-9169-0

**Published:** 2016-01-29

**Authors:** Gabriel Hattori, Jon Trevelyan, Charles E. Augarde, William M. Coombs, Andrew C. Aplin

**Affiliations:** 1 School of Engineering and Computing Sciences, Durham University, South Road, Durham, DH1 3LE UK; 2 Science Labs, Department of Earth Sciences, Durham University, Durham, DH1 3LE UK

## Abstract

Extracting gas from shale rocks is one of the current engineering challenges but offers the prospect of cheap gas. Part of the development of an effective engineering solution for shale gas extraction in the future will be the availability of reliable and efficient methods of modelling the development of a fracture system, and the use of these models to guide operators in locating, drilling and pressurising wells. Numerous research papers have been dedicated to this problem, but the information is still incomplete, since a number of simplifications have been adopted such as the assumption of shale as an isotropic material. Recent works on shale characterisation have proved this assumption to be wrong. The anisotropy of shale depends significantly on the scale at which the problem is tackled (nano, micro or macroscale), suggesting that a multiscale model would be appropriate. Moreover, propagation of hydraulic fractures in such a complex medium can be difficult to model with current numerical discretisation methods. The crack propagation may not be unique, and crack branching can occur during the fracture extension. A number of natural fractures could exist in a shale deposit, so we are dealing with several cracks propagating at once over a considerable range of length scales. For all these reasons, the modelling of the fracking problem deserves considerable attention. The objective of this work is to present an overview of the hydraulic fracture of shale, introducing the most recent investigations concerning the anisotropy of shale rocks, then presenting some of the possible numerical methods that could be used to model the real fracking problem.

## Introduction

Conventional shale reservoirs are formed when gas and/or oil have migrated from the shale source rock to more permeable sandstone and limestone formations. However, not all the gas/oil migrates from the source rock, some remaining trapped in the petroleum source rock. Such a reservoir has been named “unconventional” since it has to be fractured in order to extract the gas from inside. Hydraulic fracture, or “fracking”, has emerged as a alternative method of extracting gas and oil. Experience in the United States shows it has the potential to be economically attractive. Many concerns exist about this type of extracting operation, especially how far the fracture network will extend in shale reservoirs.

King [136] published a review paper about the last 30 years of fracking, and points out four “lessons”:

- No two shale formations are alike. Shale formations vary spatially and vertically within a trend, even along the wellbore;
- Shale “fabric” differences, combined with in-situ stresses and geologic changes are often sufficient to require stimulation changes within a single well to obtain best recovery;
- Understanding and predicting shale well performance requires identification of a critical data set that must be collected to enable optimization of the completion and stimulation design;
- There are no optimum, one-size-fits-all completion or stimulation designs for shale wells.

These points encapsulate well the uncertainties involved. Many models have been proposed over the years but they are either too simplified or they tend to focus on one key aspect of fracking (e.g. crack propagation schemes, influence of natural fractures, material heterogeneities, permeabilities). The scarcity of in-situ data makes the study of fracking even more complicated.

The most usual concerns in fracking are addressed by Soeder et al. [252], where integrated assessment models are used to quantify the engineering risk to the environment from shale gas well development. Davies et al. [55] have investigated the integrity of the gas and oil wells, analysing the number of known failures of well integrity. The modelling of reservoirs is also a difficult task due to the lack of experimental data and oversimplification of the complex fracking problem [177].

Glorioso and Rattia [97] provide an approach more focused on the petrophysical evaluation of shale gas reservoirs. Some techniques are analysed, such as log responses in the presence of kerogen, log interpretation techniques and estimation methods for different volumes of gas in-situ, among others. It is shown that volumetric analysis is imprecise for in-place estimation of shale gas; however, it is one of the few techniques available in the early stages of evaluation and development. The measurement of an accurate density of specimens is an important parameter in reducing the uncertainty inherent in petrophysical interpretations.

This paper provides an overview of the current state of fracking research. A state-of-the-art review of fracking is performed, and several points are analysed such as the models employed so far, as well as the underlying numerical methods. Special attention is given to problems involving brittle materials and the dynamic crack propagation that must be taken into account in the fracking model. The hydraulic fracture modelling problem has been tackled in several different ways, and the shale rock has mostly been assumed to be isotropic. This simplification can have serious consequences during the modelling of the fracking process, since shale rocks can present high degrees of anisotropy.

This paper is organised as follows: a description of the shale rock including the most common simplifications is presented in Sect. 2, followed by the description of the fracking operation in Sect. 3. Section 4 presents a review of the analytical formulations for crack propagation and crack branching. Different types of models such as cohesive methods and multiscale approaches are tackled in Sects. 5 and 6. Numerical aspects are discussed in Sect. 7, including the boundary element method, the extended finite element method, the meshless method, the phase-field method, the configurational force method and the discrete element method. A recently proposed discretisation method is discussed in Sect. 8. The paper ends with conclusions and a discussion of possible future research directions in Sect. 9.

## Description of the Shale Rock

Shale, or mudstone, is the most common sedimentary rock. It can be viewed as a heterogeneous, multi-mineralic natural composite consisting of sedimented clay mineral aggregates, organic matter and variable quantities of minerals such as quartz, calcite and feldspar. By definition, the majority of particles are less than 63 microns in diameter, i.e. they comprise silt- and clay-grade material. In the context of shale gas and oil, organic matter (kerogen) is of particular importance as it is responsible for the generation and, in part, the subsequent storage of oil and gas.

Mud is derived from continental weathering and is deposited as a chemically unstable mineral mixture with 70–80 % porosity at the sediment-water interface. During burial to say 200 °C and 100 MPa vertical stress, it is transformed through a series of physical and chemical processes into shale. Porosity is lost as a result of both mechanical and chemical compaction to values of round 5 % [31, 32, 287]. At temperatures above 70 °C, clay mineral transformations, dominated by the conversion of smectite to illite (e.g. [121, 254]), lead to a fundamental reorganisation of the clay fabric, converting it from a relatively isotropic fabric to one in which the clay minerals are preferentially aligned normal to the principal (generally vertical) stress [56, 57, 120]. Although quantitative mechanical data are scarce for mineralogically well-characterised samples, it is likely that the clay mineral transformations strengthen shales [206, 264]. In muds which contain appreciable quantities of biogenic silica (opal-A) and calcite, the conversion of opal-A to quartz [134, 281], and dissolution-reprecipitation reactions involving calcite [259], will also strengthen the shale. Indeed, it is generally considered that fine-grained sediments which are rich in quartz and calcite are more attractive unconventional oil and gas targets compared to clay-rich media, as a result of their differing mechanical properties (e.g. [204]).

Shales with more than ca. 2 % organic matter act as sources and reservoirs for hydrocarbons. Between 100 and 200 °C kerogen is converted to hydrogen-rich liquid and gaseous petroleum, leaving behind a carbon-rich residue (e.g. [126, 144, 207]). The kerogen structure changes from more aliphatic to more aromatic, and its density increases [194]. Changes in the mechanical properties of kerogen with increasing maturity are not well documented. However, they may be quite variable, depending on the nature of the organic matter. For example, Eliyahu et al. [68] performed PeakForce QNM® tests with an atomic force microscope to make nanoscale measurements of the Young’s modulus of organic matter in a single shale thin section. Results ranged from 0-25 GPa with a modal value of 15 GPa.

Shales are heterogeneous on multiple scales ranging from sub-millimetre to tens of metres (e.g. [10, 204]). Hydrodynamic processes associated with deposition often result in a characteristic, ca. millimetre-scale lamination [35, 157, 241], which can be disturbed close to the sediment-water interface by bioturbation [63]. On a larger, metre-scale, parasequences form within mud-rich sediments, driven by orbitally-forced changes in climate, sea-level and sediment supply [35, 156, 157, 204]. Parasequence boundaries are typically defined by rapid changes in the mineralogy and grain size of mudstones, with more subtle variations within the parasequence. Stacked parasequences add further complexity to the shale succession and result in a potentially complex mechanical stratigraphy which depends on the initial mineralogy of the chosen unit and the way that burial diagenesis has altered physical properties on a local scale.

During the shale formation process bedding planes are formed, which may present sharp or gradational boundaries. This is the most regular type of deposition that occurs in shales. Deposition may not be uniform during the whole process, presenting discontinuities at some points or other type of deposition patterns. This makes the mechanical characterisation of shale a complex issue. Moreover, not all shale rocks are the same, so a prediction made for an specific shale rock probably is not valid elsewhere.

The works of Ulm and co-workers about nanoindentation in shale rocks [34, 198–200, 268–270] have been important developments in our ability to characterise the mechanical properties of shale rocks. From [268], it is seen that shales behave mechanically as a nanogranular material, whose behaviour is governed by contact forces from particle-to-particle contact points, rather than by the material elasticity in the crystalline structure of the clay minerals. This assumption is valid for scales around 100 nm.

The indentation technique consists of bringing an indenter of known geometry and mechanical properties (typically diamond) into contact with the material for which the mechanical properties are to be known. Through measurement of the penetration distance *h* as a function of an increasing indentation load *P*, the indentation hardness *H* and indentation modulus *M* are given by

1 $$H= \frac{P}{A_c}$$

2 $$M= \frac{\sqrt{\pi }}{2} \frac{S}{\sqrt{A_c}}$$

where $A_c$ is the projected area of contact and $S = (dP/dh)_{h_{max}}$ is the unloading indentation stiffness. For the case of a transversely isotropic material, where $x_3$ is the axis of symmetry, the indentation modulus is given by [268, 269]

3 $$M_3 = \sqrt{2\left( \frac{C_{31}^2-C_{13}^2}{C_{11}}\right) \left( \frac{1}{C_{44}}+\frac{2}{C_{31}+C_{13}}\right) ^{-1} }$$

and for the $x_1,x_2$ axis by

4 $$M_1 = M_2 = \sqrt{\frac{C_{11}^2-C_{12}^2}{C_{11}}\sqrt{\frac{C_{11}}{C_{33}}}M_3 }$$

where $C_{ij}$ come from the constitutive matrix and are given in the Voigt notation [276].

From [269], it was seen that the level of shale anisotropy increases from the nanoscale to the macroscale. Macroscopic anisotropy in shale materials results from texture rather than from the mineral anisotropy. The multiscale shale structure can be divided into 3 levels:

1. Shale building block (level I - nanoscale): composed of a solid phase and a saturated pore space, which form the porous clay composite. A homogeneous building block, which consists in the smallest representative unit of the shale material, is assumed at this scale. The material properties are composed of two constants for the isotropic clay solid phase, the porosity and the pore aspect ratio of the building block.
2. Porous laminate (level II - microscale): the anisotropy increases due to the particular spatial distribution of shale building blocks (considering different types of shale rocks). The morphology is uniform allowing the definition a Representative Volume Element (RVE).
3. Porous matrix-inclusion composite (level III - macroscale): shale is composed of a textured porous matrix and (mainly) quartz inclusions of approximately spherical shape that are randomly distributed throughout the anisotropic porous matrix. The material properties are separated into six indentation moduli plus the porosity.

One can observe that the heterogeneities are manifested from the nanoscale to the macroscopic scale, and combine to cause a pronounced anisotropy and large variety in shale macroscopic behaviour.

Nanoindentation results provide strong evidence that the nano-mechanical elementary building block of shales is transversely isotropic in stiffness, and isotropic and frictionless in strength [34]. The contact forces between the sphere-like particles activate the intrinsically anisotropic elastic properties within the clay particles and the cohesive bonds between the clay particles.

The determination of the mechanical microstructure and invariant material properties are of great importance for the development of predictive microporomechanical models of the stiffness and strength properties of shale.

## The Hydraulic Fracturing Process and Its Modelling

The hydraulic fracture or fracking operation involves at least three processes [3]:

1. The mechanical deformation induced by the fluid pressure on the fracture surfaces;
2. The flow of fluid within the fracture;
3. The fracture propagation

The shale measures in question are usually found at a distance of 1 to 3 km from the surface. A major concern relating to fracking is that the fracture network may extend vertically, allowing hydrocarbons and/or proppant fluid to penetrate into other rock formations, eventually reaching water reservoirs and aquifers that are found typically approximately 300 m below the surface.

Fracking can occur naturally, such as in magma-driven dykes for example. In the 1940s, when fracking started commercially in US, the hydraulic fracture was applied through a vertical drilling. In that case, the pressurised liquid was applied perpendicular to the bedding planes. It was known that the shale was a layered material due to its formation process, but technology of that time was very limited.

**Fig. 1:**
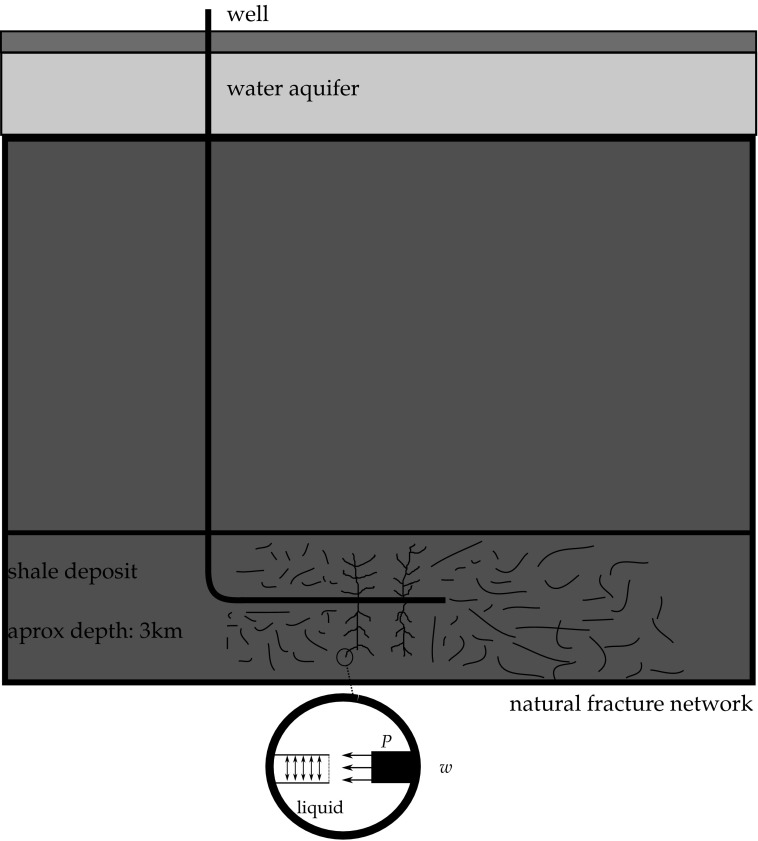
Fracking example

In the last 15 years, recent engineering advances have allowed engineers to change the direction of the drilling, making it possible to drill a horizontal well and consequently, to pressurise the shale rocks in the same horizontal plane of the bedding plane, making the fracking process much more effective. Figure 1 illustrates the structure of the well’s drilling, and the natural fracture network that can be found. In detail it is a sketch of the pressurised liquid entering a crack, resulting in the application of a pressure *P* over the crack surfaces and the crack opening *w*.

The horizontal drilling was not new to the industry, but it was fundamental for the success of shale gas developments. From 1981 to 1996, only 300 vertical wells were drilled in the Barnett shale of the Fort Worth basin, north central Texas. In 2002, horizontal drilling has been implemented, and by 2005 over 2000 horizontal wells had been drilled [40]. The Barnett shale formation found in Texas produces over 6 % of all gas in continental United States [273]. The application of this new drilling technique has turned the United States from a nation of waning gas production to a growing one [221].

To optimise the fracking process of shale, it is important to detect accurately the location of natural fractures. The anisotropic behaviour of the shale generates preferential paths through the shale fabric [136, 279]. Moreover, the alignment of the natural fractures can also induce anisotropic patterns of the fluid flow [86, 87].

### Modelling of the Shale Fracture

Much of the work done so far in attempting to model shale fracture is very simple, taking into account only the influence of the crack and not the fluid. Only recently have a few researchers [3, 4, 62, 160, 161, 188, 205, 296] successfully developed more sophisticated methods including the fluid-crack interaction.

The usual assumption in hydraulic fracture is that the fracture is embedded within an infinite homogeneous porous medium, where flow occurs only perpendicular to the fracture plane, which was first defined by Carter [46]. Moreover, the injection pressure does not propagate beyond the current extent of the fracture. Carter’s model can lead to an overestimate of the fracture propagation rate by a factor of 2 as compared to a 3D model [161]. The reason is that the pressure increases beyond the length of the hydraulic fracture, causing an increasing of the leak-off and a corresponding reduction in fracture growth. The leak-off rate $Q_1$ is given as [161]

5 $$\left. Q_1 = -4\pi \frac{k_z}{\mu } \int _0^{a(t)} r \frac{\partial P}{\partial z}\right| _{z=0}\ dr$$

where $\mu$ is the fluid viscosity, $k_\alpha$ is the permeability in the $\alpha$-direction ($\alpha = r$ or $\alpha = z$), *P* is the hydraulic pressure, *a*(*t*) is the hydraulic fracture radius, dependent of time *t*, *r* and *z* are the distances parallel and normal to the fracture plane, respectively. The hydraulic pressure is defined by the boundary value problem

6 $$S \frac{\partial P}{\partial t} = \frac{k_r}{\mu }\frac{1}{r}\frac{\partial }{\partial r} \left( r \frac{\partial P}{\partial r} \right) + \frac{k_z}{\mu }\frac{\partial ^2 P}{\partial z^2}$$

where *S* is a storage coefficient of the porous medium. The solution of Eq. (6) can be obtained using a standard finite volume method, as used in [161].

Assuming that the faces of the fracture are loaded by a uniform pressure $P_d$, the displacement of the fracture face normal to the fracture plane $\delta$ is given by

7 $$\delta = \frac{4(1-\upsilon ^2)P_d a}{\pi E}\left[ 1-\left( \frac{r}{a} \right) ^2 \right] ^{1/2}$$

where $\upsilon$ is the Poisson’s ratio and *E* is the Young’s modulus.

The fracture volume *V* is found from

8 $$V = 4\pi \int _0^a r \delta (r)\ dr = \frac{16(1-\upsilon ^2)P_d a^3}{3E}$$

The energy release rate *G* of the rock is obtained from the following expression

9 $$G = \frac{P_d}{2a} \int _0^a r \frac{d\delta }{da} dr = \frac{2(1-\upsilon ^2)P_d^2 a}{\pi E}$$

and is related to the mode I stress intensity factor $K_{I}$ through the expression

10 $$G = \frac{K_{I}^2}{E}$$

From Eqs. (8) and (9), it is possible to write $P_d$ and *a* in terms of *V* as

11 $$P_d= \frac{3}{16} \left[ \left( \frac{256\pi G}{18}^3 \right) ^3 \left( \frac{E}{1-\upsilon ^2} \right) ^2 \right] ^{1/5} V^{-1/5}$$

12 $$a= \left[ \frac{18}{256\pi G} \left( \frac{E}{1-\upsilon ^2} \right) \right] ^{1/5} V^{2/5}$$

In the early stages following initial pressurisation, the volume of injected fluid is sufficiently small such that the the porous formation do not absorb the incoming fluid. As injection process continues, the fluid is accommodated locally in the pore space and consequently predicts leak-off. Once the system reaches steady-state, it again becomes independent of porosity system. This analytical formulation have issues when predicting the behaviour during the transient state [161].

Even though these models can represent complex processes occurring during fracking, they are still far from being accurate, mainly because shale is considered to be isotropic, which has been seen not to be true [268], and since the material presents nanoporosity, it is difficult to accurately model the mechanical properties of shale.

Some other open questions are [3]:

- how to appropriately adjust current (linear elastic) simulators to enable modelling of the propagation of hydraulic fractures in weakly consolidated and unconsolidated “soft” sandstones;
- laboratory and field observations demonstrate that mode III fracture growth does occur, and this needs to be further researched.

Some works have analysed the crack propagation path in shales, including refracturing sealed wells. For example, Gale and co-workers [87] found that propagation of the hydraulic fracture over a natural fracture will cause delamination of the cement wall and the shale. The fluid enters the fracture and causes further opening of the fracture in a direction normal to the propagating hydraulic fracture while the pressure inside the fracture increases. After the fracture propagation at the natural fracture reaches a sealed fracture tip, the hydraulic fracture resumes growth parallel to the direction of maximum shear stress.

In an analytical work, Vallejo [271] has investigated the hydraulic fracture on earth dam soils, where shear stresses were seen to promote crack propagation on traction free cracks. Other analytical study about re-fracturing was carried out by [295], where the dynamic fracture propagation is characterised in low-permeability reservoirs. The results are comparable to an experimental test with the same material parameters.

In summary, research works in hydraulic fracture formulation have considered a large number of variables and processes which occur during the actual operation: leak-off, shale permeabilities, crack opening and fluid interaction over a crack surface. However, the current analytical theories for hydraulic fracture do not include crack propagation conditions, especially dynamic crack propagation, neither crack branching, since material instabilities at the crack tip during crack propagation may cause the propagation path not to be unique. These concerns are summarised in the next section.

## Crack Propagation and Crack Branching

Consider a homogeneous isotropic body under a known applied loading. The resulting elastic stress distribution over the body due to the applied force is generally smooth. However, introducing a discontinuity such as a crack imposes a singular behaviour to the stress distribution. It can be shown that the stress increases as it is measured closer to the crack tip, varying with $1/\sqrt{r}$, where *r* is the distance from the crack tip. Irwin [125] proposed that the asymptotic stress field at the crack tip is governed by parameters depending on the geometry of the crack and the applied load. These parameters are known as Stress Intensity Factors (SIFs) and have been widely used as criteria for crack stability and propagation. The three SIFs, $K_I, K_{II}, K_{III}$, each correspond to one of three modes of crack behaviour: mode I (opening), mode II (sliding) and mode III (tearing). In this paper we will confine ourselves mostly to mode I.

It can be postulated that crack growth will begin if the value of the SIFs increase to a certain value. If the SIF is higher than a critical fracture toughness parameter $K_c$, which depends on the material properties, then the crack will propagate through the body. The situation becomes more complicated when the load is applied rapidly so that dynamic effects become important. This does not imply that the value of the dynamic fracture toughness will be independent of the rate of loading or that dynamic effects do not influence the fracture resistance in other ways [82].

In some cases, the toughness appears to increase with the rate of loading whereas in other cases the opposite dependence is found. The explanation for the shift must be sought in the mechanisms of inelastic deformation and material separation in the highly stressed region of the edge of the crack in the loaded body [82]. The dynamic crack propagation formula can be defined as

13 $$K_I^d = \kappa (v)\ K_I^s(a)$$

where $K_I^d$ is the mode I dynamic SIF, $K_I$ is the static SIF, *v* is the crack velocity and $\kappa (v)$ is a scaling factor. When $\kappa (v) = 1$, the crack velocity is zero, whereas $\kappa (v)=0$ indicates that the crack velocity is equal to the Rayleigh wave speed.

The theoretical limiting speed of a tensile crack must be the Rayleigh wave speed. This was anticipated by Stroh [257] on the basis of a very intuitive argument [82].

Gao et al. [89] studied crack propagation in an anisotropic material, and presented expressions for the dynamic stresses and displacements around the crack tip. These predict that larger crack propagation velocities induce higher stress and displacement fields at the crack tip. The limiting speed in crack propagation is analysed in [88], where a local wave speed resulting from the elastic response near the crack tip also changes with the crack propagation velocity. A molecular dynamic model is used in this work, so crack propagation is modelled as bond breakage between the particles. The crack velocity is expressed using the Stroh formalism.

There are three types of criteria for brittle crack propagation:

1. Maximum tangential stress: This criteria was defined by Erdogan and Sih [69] and is based in two hypothesis:
  - The crack extension starts at its tip in radial direction;
  - The crack extension starts in the plane perpendicular to the direction of greatest tension.
  The crack propagates when the SIF is higher than a critical SIF $K_c$, which depends on the materials properties. From [69], the crack propagation angle $\theta _p$ can be obtained from the following relation
  14 $$K_I \sin {\theta _p} + K_{II}(3\cos {\theta _p}-1)=0$$
  where $K_I$ and $K_{II}$ are the mode I and mode II SIF, respectively, and $\theta _p$ is taken with respect to the horizontal axis. This crack propagation criteria was extended to anisotropic materials in [239].
2. Strain energy release rate: In this criteria, the crack propagates when energy release rate *G* reaches some critical value $G_c$, taking the direction where *G* is maximum [123]. The energy release rate is defined as
  15 $$G = \frac{\partial W}{\partial a}$$
  where *W* represents the strain energy and *a* is the half-crack length. Equation (15) can be expressed in terms of mixed mode SIFs for an isotropic material as
  16 $$G = \frac{1-\upsilon ^2}{E}\left( K_I^2 + K_{II}^2 \right) + \frac{1}{2\mu }K_{III}^2$$
  where $\mu$ is the shear modulus.
3. Minimum strain energy: crack propagation occurs at the minimum value of the strain density *S* defined as [169, 247]
  17 $$S = a_{11}K_I^2 + 2a_{12}K_I K_{II}+a_{22}K_{II}^2+a_{33}K_{III}^2$$
  where $a_{ij}$ come from the material properties. The direction of propagation goes toward the region where *S* assumes a minimum value $S_{min}$. The crack extension $r_0$ is proportional to the minimum strain energy, such that the ratio $\frac{S_{min}}{r_0}$ is constant along the crack front [169].

One can observe that all these criteria are related to the SIFs. These criteria are well consolidated in the fracture mechanics literature over the years. However they fail in one aspect, since they do not consider the possibility of crack branching, i.e., at some point of the crack propagation process, the crack may bifurcate in two or more new cracks. This issue is especially important when modelling highly heterogeneous materials such as the shale rock.

Yoffe [289] attempted to explain the branching of cracks from an analysis of the problem of a crack of constant length that translates with a constant velocity in an unbounded medium. From this solution she found that the maximum stress acted normal to lines that make an angle of $60^\circ$ with the direction of crack propagation when the crack velocity exceeded 60 % of the shear wave speed. This fact might cause the crack to branch whenever the crack velocity exceeds that value. However, Yoffe did not consider that the maximum stress would be perpendicular to the crack path, so this assumption is not valid for brittle materials. Moreover, the $60^\circ$ angles are quite large in comparison with the branching angles observed from experiments [223].

Ravi-Chandlar and Knauss [222, 223] have addressed the crack propagation and crack branching problems through several experiments. From [222], the crack branching has the following properties

1. crack branching is the result of many interacting microcracks or microbranches;
2. only a few of the microbranches grow larger while the rest are arrested;
3. the branches evolve from the microcracks which are initially parallel to the main crack, but deviate smoothly from the original crack orientation;
4. the microbranches do not span the thickness of the plate, some occurring on the faces of the plate while others are entirely embedded in the interior of the plate.

Sih [247] made the hypothesis that the instability that occurs in crack bifurcation is associated with the fact that a high speed crack tends to change its direction of propagation when it encounters an obstacle in the material. The excess energy in the vicinity of such a change in direction is sufficient to initiate a new crack. This event occurs so quickly that the crack appears to have been split in two, or bifurcated.

From [223], one can see that the velocity with which the crack propagates is determined by the SIF at initiation. Cracks propagating at low speeds may undergo a change in the crack velocity if stress waves are present. Cracks propagating at high speeds do not change crack velocity, but may exhibit crack branching.

Crack branching formulations can be found in [78, 131, 247, 289], to cite just a few works. In all cases, only the isotropic material case is considered. For anisotropic crack branching, numerical methods have to be employed.

## Cohesive Methods

The fracture process is usually considered only at the crack tip. In such cases, the fracture process zone is considered to be small compared to the size of the crack [17, 66, 67].

In Linear Elastic Fracture Mechanics (LEFM), the stress becomes infinite at the crack tip. Since no material can withstand such high stress, there will be a plastification/fracture zone around the crack tip.

The fracture process zone can be described by two simplified approaches [178]:

1. The fracture process zone is lumped into the crack line and is characterised as a stress-displacement law with softening;
2. Inelastic deformations in the process zone are smeared over a band of a given width, imagined to exist in front of the main crack.

Most of the work done in cohesive cracks makes use of the former approach, otherwise known as the Dugdale–Barrenblatt model, fictitious crack model or stress bridging model [178].

**Fig. 2:**
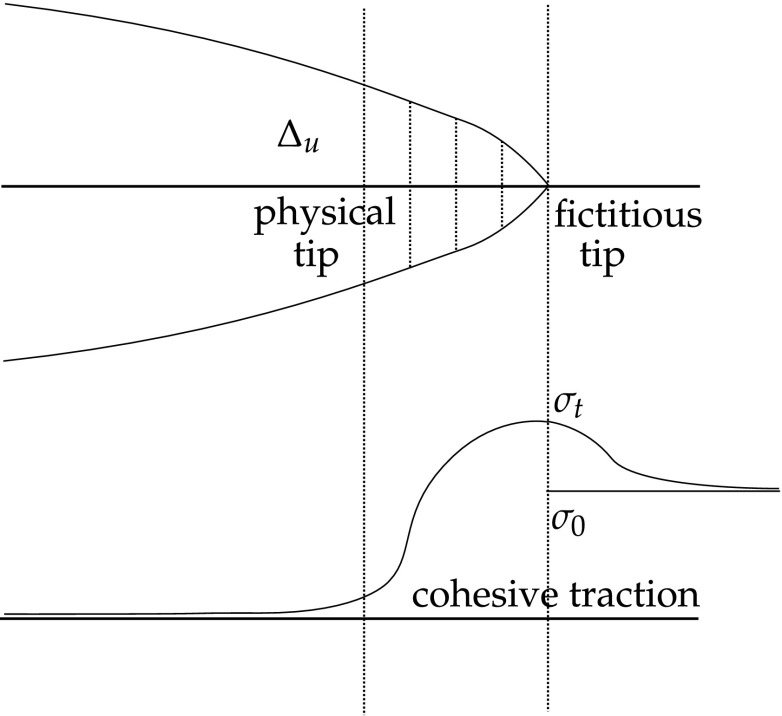
Cohesive crack

The non-linear behaviour around the crack tip can be considered to be confined to the fracture process zone on the crack surface. Figure 2 illustrates a crack with its corresponding fracture process zone. One can see two tips in this model, the physical tip, where the tractions vanish, and the fictitious tip, where the displacement is zero.

Since there is no singularity at the crack tip, the SIF should vanish. This condition is also called the zero stress intensity factor, and is represented by the superposition of two states

18 $$K_I^{phys} + K_I^{fict} = 0$$

where $K_I^{phys}$ corresponds to the SIF at the physical crack tip, and $K_I^{fict}$ is the SIF at the fracture process zone. Here we consider only the mode I fracture without loss of generality.

**Fig. 3:**
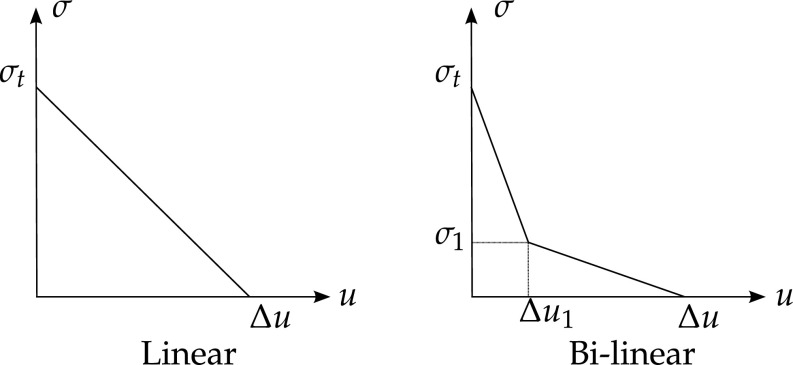
Relation between stress and relative displacement at the crack faces

The crack propagates when the maximum principal stress reaches the material tensile strength $\sigma _t$, so fracture is initiated at the fracture process zone. The stress on the crack faces depends directly on the relative displacement $\Delta u$ of the crack faces [213]. There are different types of stress-displacement functions which model the behaviour in the fracture process zone. Figure 3 presents two of the most common assumptions

Dugdale [66] and Barenblatt [17] models are the basis of many cohesive models. The Dugdale cohesive crack model is very simplistic and is best used for ductile materials. A uniform traction equal to the yield stress is used to describe the softening in the fracture process zone.

Most of the cohesive models are developed for isotropic materials (see [67] for example). However, there are some models for heterogeneous materials [11, 216, 244] and composite [148, 181, 261, 272] materials. Nevertheless, the material models are quite simple, usually considering different types of isotropic materials instead of a full anisotropic model. To the authors’ best knowledge, there is no anisotropic cohesive crack model to this date.

Cohesive models have been also applied in multiscale problems, where cracks are significantly smaller than the RVE. In [210], a microelastic cohesive model is developed for quasi-brittle materials. The stability of crack growth is analysed, and it is concluded that macroscopic strength is not necessarily correlated to crack propagation, and may be caused by unstable growth of cohesive zones ahead of non-propagating cracks. The initial cohesive zone has a significant influence on the macrostrength of quasi-brittle materials.

A number of different approaches for cohesive models have been proposed over the years. Enriched formulations for delamination problems were analysed by Samimi [236–238]. A stochastic approach for delamination in composite materials was proposed in [181], where the imperfections of the material were considered in the cohesive model. The cohesive crack has been extensively studied as can be seen in [59, 75, 102, 152, 209, 297] to cite just a few works. Crack propagation in cohesive models was recently discussed in [152, 298] for example.

A rudimentary model for hydraulic fracture for isotropic materials using the finite element software ABAQUS was considered in [288]. Papanastasiou [203] has evaluated the fracture toughness in hydraulic fracturing, modelling the rock-fluid coupling through a finite element model with cohesive behaviour. The Mohr-Coulomb criterion is used to take plasticity into account in the rock deformation. The plastic behaviour that develops around the crack tip provides an effective shielding, resulting in an increase in the effective fracture toughness.

## Multiscale

**Fig. 4:**
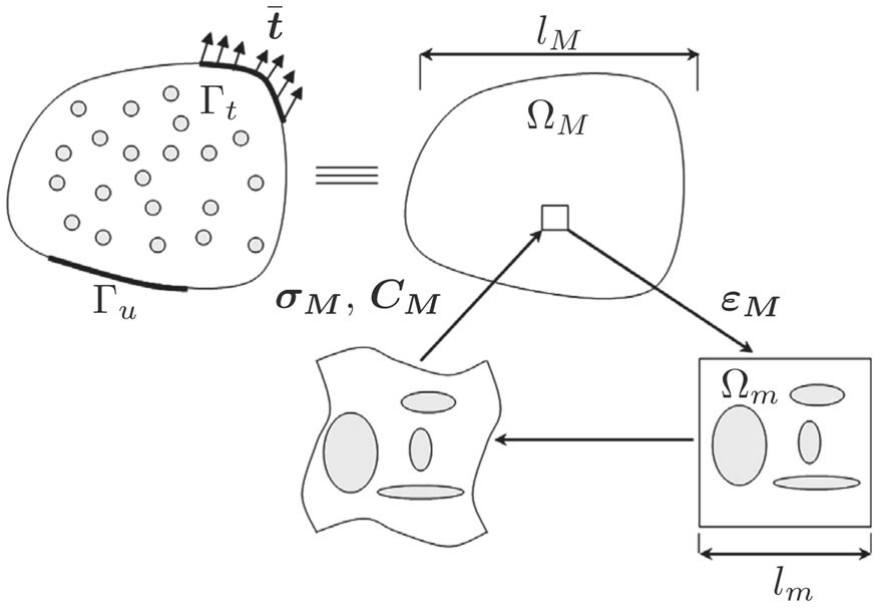
Scheme of the choice of a representative volume element (RVE) (From [130])

The main advantage of multiscale models is to make different hypotheses at different levels within the same problem. For example, material can present distinct degrees of anisotropy depending on the scale of observation (nano, micro or macro). The coupling of different scales can be cumbersome. Some sort of regularisation is commonly used to enforce the coupling between scales. A typical assumption is the use of a Representative Volume Element (RVE), a representative part of the model at the reduced scale so it contains all the distinct properties of the considered scale, and is also defined as the local model. The global model takes the RVE as a homogenised representation of the material’s properties at the large scale. An example of an RVE is illustrated in Fig. 4.

Another important part of multiscale modelling is the coupling of stresses and strains from the local and global models. Numerical homogenisation is a popular technique and is an alternative to the traditional analytical homogenisation. It is especially used for monophasic heterogeneous materials, where the balance and constitutive equations are considered at the RVE level. The first work in numerical homogenisation is due to Ghosh et al. [96].

Zeng et al. [292] proposed a multsicale cohesive model for geomaterials. At the macroscopic scale, a sample of polycrystalline material is considered as a continuum made of many material points. The estimation of the material properties at the microscale is performed by statistical homogenisation, since the RVE represents a number of different constituents or phases, as mineral grains and voids, and is therefore composed of randomly distributed constituents.

**Fig. 5:**
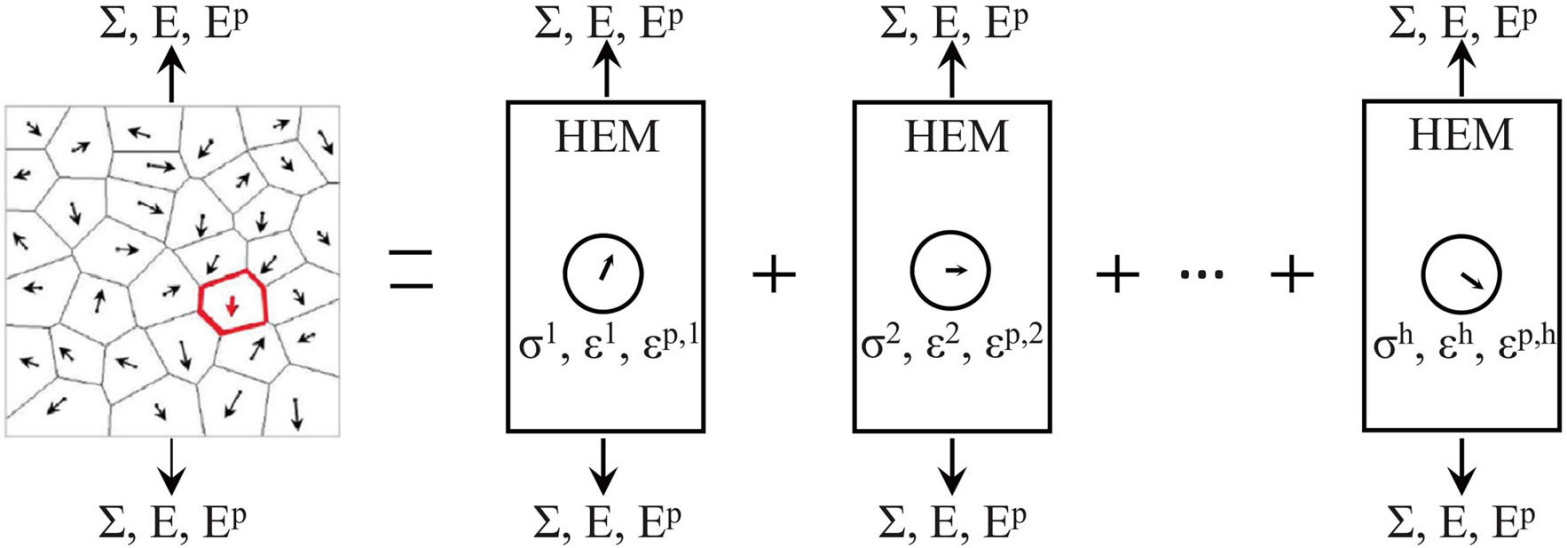
A homogeneous equivalent medium (HEM) scheme (from [292])

The Eshelby elastic solution for the spherical inclusion problem [71, 72] is used to obtain the local stress and strain fields. Therefore, the strains or stresses in a single crystal are approximated by considering a spherical single crystal embedded in an infinite elastically deformed matrix. The KBW model, named after Kröner [140], Budiansky and Wu [43], extends Eshelby’s formulation by taking into account the grain interaction and plastification. By the KBW definition, each crystal is embedded in a Homogeneous Equivalent Medium (HEM) as shown in Fig. 5.

The local stress $\varvec{\sigma }$ and strain $\varvec{\varepsilon }$ are related to the global stress $\varvec{\Sigma }$ and strain $\varvec{E}$ as follows

19 $$\varvec{\sigma }- \varvec{\Sigma }= - {\mathscr {L}} (\varvec{\varepsilon }- \varvec{E})$$

where ${\mathscr {L}}$ is the interaction tensor and is given by

20 $${\mathscr {L}} = {\mathbf{M}} ({\mathbf{S}}^{-1} - {\mathbf{I}})$$

where ${\mathbf{M}}$ is the homogenised elastoplastic tangent operator of HEM, ${\mathbf{S}}$ is the Eshelby’s tensor and ${\mathbf{I}}$ is a third order identity matrix.

Zeng and Li [293] developed a multiscale cohesive zone method, where the local fields are determined through measures of the bond at the atom particle level. The stress relation coupling the local and global fields is given by

21 $$\varvec{\sigma }= \frac{1}{\Omega ^b} \sum _{i=1}^{n_b} \frac{\partial \phi }{\partial r_i} \frac{{\mathbf{r}}_i \otimes {\mathbf{r}}_i}{r_i}$$

where $\Omega ^b$ is the volume of the unit cell, $n_b$ is the total number of bonds in a unit cell, $\phi (r_i)$ is the atomistic potential, $r_i,i=1,\cdots , n_b$ is the current bond length for the *i*th bond in a unit cell and is given by ${\mathbf{r}}_i = {\mathbf{F}}_e {\mathbf{R}}_i$, with the deformation gradient ${\mathbf{F}}_e$ in element *e* and the underformed bond vector ${\mathbf{R}}$. The symbol $\otimes$ denotes the outer, or dyadic, product.

The strain energy in a given element $\Omega _e$ can be written as

22 $$E_e = \frac{1}{\Omega _0^b}\sum _{i=1}^{n_b}\phi (r_i({\mathbf{F}}_e)) \Omega _e= W({\mathbf {F}}_e) \Omega _e$$

and therefore the total energy is defined as

23 $$E_{tot} = \sum _{\alpha =1}^{N_{rep}} n_\alpha E_\alpha ({\mathbf {u}}_\alpha )$$

where $n_\alpha$ is a chosen weight and $\sum n_\alpha =1$. The energy from each representative atom $E_\alpha$ is obtained by the interaction with the neighbouring atoms whose positions are generated using the local deformation.

This formulation is referred to as the local QC method, a simplification of the continuum system when interface and surface energies may be neglected. The general non-local QC potential energy may lead to some non-physical effects in the transition region. The derivatives of the energy functional to obtain forces on atoms and finite element nodes may lead to so-called ghost forces in the transition region between the macro and microscale, and it has several issues that remain to be resolved, such as the computation of approximations in the macroscale far from microscale defects [245] and the correct balance of energy which needs to be ensured between macro and microscales [174].

Since the connections between atoms are modelled through bonds, this multiscale cohesive formulation is able to capture the crack branching behaviour during crack propagation.

In [299], the RVE properties of a hydrogeologic reservoir are averaged through statistical parameters. The main reason is that the heterogeneity of the reservoir can be more easily modelled through the mean and standard deviation of the rock properties. The site scale represents the entire solution domain used for modelling global flow and transport. The layer scale represents geologic layering in the vertical direction. Within a layer, relatively uniform properties are present in both vertical and lateral direction, in comparison with the larger variations between different layers that may vary significantly in thickness. The local scale represents the variation of properties within a hydrogeologic layer.

In [164], a multiscale model for the shale porous network is proposed. Permeability is assumed as an intrinsic porous medium property independent of fluid properties (such as viscosity) or thermodynamic conditions. The porous medium was modelled as networks of pores connected by throats. This simplification neglects the physics of the real porous network. Permeability further depends on the relative size of the void spaces as well as the fraction of pores belonging to each length scale. Unlike absolute permeability in conventional reservoirs, gas permeability depends on absolute pressure values in individual pores (and not only the gradient). Specifically, smaller pressures result in (somewhat counter-intuitively) an increase in permeability.

A number of multiscale models for brittle materials can be mentioned: [2, 83, 130, 209, 274] just to cite some of the most recent works.

## Discrete Numerical Methods

In this section we will present a brief description of different element-based numerical methods that can be used in the modelling of fracking problems. The boundary element method (BEM) has been used in brittle anisotropic problems including crack propagation. The extended finite element method (X-FEM) has been developed recently and is also a good choice for fracture mechanics problems, and can be easily applied in cohesive models. Meshless methods are becoming popular in fracture mechanics problems. The discrete element method (DEM) is particularly used in problems with rock materials. The phase-field method and the configurational force method are also reviewed in this section.

### Boundary Element Method (BEM)

The boundary element method has first appeared in the work of Cruse and Rizzo [52] for elasticity problems, but it was effectively named as BEM in the work of Brebbia and Domínguez [41] and represented a series of advances in comparison to the existent domain discretisation methods as the finite element method (FEM) and the finite differences method (FDM) [109]:

- Accurate mathematical representations of the underlying physics are employed, resulting in the ability of the BEM to provide highly accurate solutions;
- The problem is defined only at the boundaries, which gives a reduction of dimensionality in the mesh (linear for 2D problems and surface for 3D problems), therefore resulting in a reduced set of linear equations to be solved;
- In spite of the boundary-only meshing, results at any internal point in the domain can be calculated once the boundary problem has been solved;
- There is a great advantage in certain classes of problem that can be characterised by either (1) infinite (or semi-infinite) domains, or (2) discontinuous solution spaces. These advantages have resulted in the BEM gaining popularity for acoustic scattering, fracture mechanics, re-entry corners and other stress intensity problems, where domain discretisation methods have poorer convergence.

However, there are some drawbacks which may deterred FEM users from migrating to the BEM:

- The system of equations is both non-symmetric and fully populated, which may lead to longer computing times (compared to FEM for example), especially in 3D problems. In this case, techniques such as the fast multipole method [228] have been introduced to accelerate the solution in large-scale problems;
- A Fundamental Solution (FS) or Green’s function, describing the behaviour of a point load in an infinite medium of the material properties is required as part of the kernel of the method. This can make the use of BEM infeasible in problems where a FS is not available;
- calculation of the FS must be computationally efficient, which makes explicit FS formulations very desirable in this sense. Dynamic problems usually have implicit formulations, see [60, 227, 277] for instance, where the FS is expressed in a integral form by means of the Radon transform;
- The BEM formulation requires the evaluation of weakly singular, strongly singular and sometimes hypersingular integrals which must be carefully treated. This can be done through a variety of methods, including singularity substraction, e.g. [100], or analytical regularisation, e.g. [91];
- Non-linear problems (e.g. material non-linearities) are difficult to model;

The constitutive equations are given as

24 $$\sigma _{ij}= C_{ijkl}\epsilon _{kl}$$

with $C_{ijkl}$ and $\sigma _{ij}$ denoting the elastic stiffness and the mechanical stresses, respectively, and

25 $$\epsilon _{ij} = \frac{1}{2}\left( u_{i,j} + u_{j,i} \right)$$

where $u_i$ are the elastic displacements. The Einstein summation notation applies in Eqs. (24) and (25).

The elastic tractions $p_{ij}$ are given by

26 $$p_{i}=\sigma _{ij}n_{j}$$

with ${\mathbf {n}} = (n_1,n_2,n_3)$ being the outward unit normal to the boundary.

The time-harmonic equilibrium equations in the absence of body forces can be written as

27 $$\sigma _{ij,j}({\mathbf {x}},t) + \rho \ddot{u}_i({\mathbf {x}},t) = 0$$

where *t* is the time and $\rho$ is the mass density of the material.

**Fig. 6:**
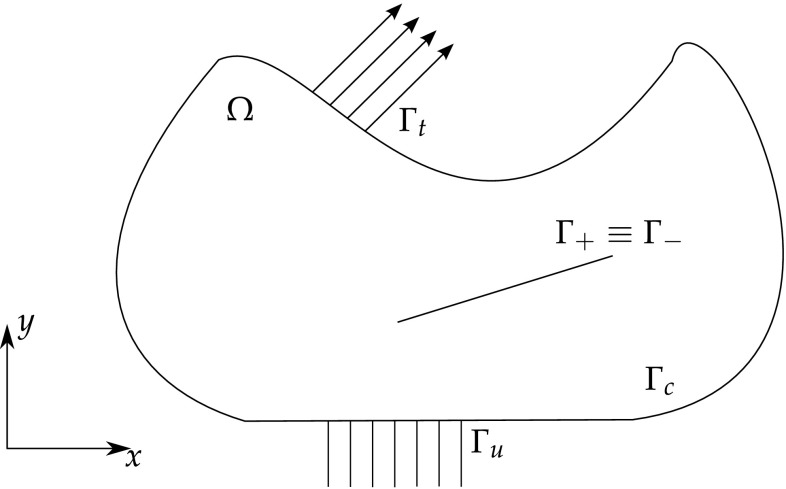
Elastic body with a crack

From Fig. 6, let $\Omega$ be a cracked domain with boundary $\Gamma$, which can be decomposed into two boundaries, an external boundary $\Gamma _c$ and an internal crack $\Gamma _{crack} = \Gamma _+ \cup \Gamma _-$ represented by two geometrically coincident crack surfaces.

The Dual BEM formulation for time-harmonic loading relies on two boundary integral equations (BIEs), one with respect to the displacements at a point $\varvec{\xi }$ of the domain $\Omega$

28 $$c_{ij}(\varvec{\xi })u_{j}(\varvec{\xi },t)+\int _{\Gamma } p_{ij}^{*}(\varvec{x},\varvec{\xi },t)u_{j}(\varvec{x},t )\ d\Gamma (\varvec{x})=\int _{\Gamma }u_{ij}^{*}(\varvec{x} ,\varvec{\xi },t)p_{j}(\varvec{x},t)\ d\Gamma (\varvec{x} )$$

and a BIE with respect to the generalised tractions

29 $$c_{ij}(\varvec{\xi })p_{j}(\varvec{\xi },t) +N_{r}\int _{\Gamma }s_{rij}^{*}(\varvec{x},\varvec{\xi },t)u_{j}(\varvec{x},t )\ d\Gamma (\varvec{x}) =N_{r}\int _{\Gamma }d_{rij}^{*}(\varvec{x},\varvec{\xi },t )p_{j}(\varvec{x},t)\ d\Gamma (\varvec{x})$$

which follows from the differentiation of the displacement BIE and further substitution into the constitutive laws equation (for details see [90]). $N_r$ stands for the outward unit normal to the boundary at the collocation point $\varvec{\xi }, c_{ij}$ is the free term that comes from the Cauchy Principal Value integration of the strongly singular kernels $p_{ij}^{*}, u_{ij}^*$ and $p_{ij}^{*}$ are the displacement and traction FS and $d_{rij}^*$ and $s_{rij}^*$ follow from derivation and substitution into Hooke’s law of $u_{ij}^*$ and $p_{ij}^{*}$, respectively.

In most cases, the cracks are considered to be free of mechanical tractions. These boundaries conditions can be summarised as

30 $$\Delta p_j = p_j^+ + p_j^- = 0$$

where the ‘+’ and ‘−’ superscripts represents the upper and lower crack surfaces, respectively. Eqs. (28) and (29) can be redefined in terms of the crack tip opening displacement ($\Delta u_{J}=u_{J}^{+}-u_{J}^{-}$) in function of the crack-free boundary $\Gamma_c$ and one of the crack surfaces, say $\Gamma _+$

31 $$\begin{aligned}&c_{ij}(\varvec{\xi })u_{j}(\varvec{\xi },t)+\int _{\Gamma _{c}}p_{ij}^{*}(\varvec{x},\varvec{\xi },t) u_{j} (\varvec{x},t)\ d\Gamma (\varvec{x}) + \int _{\Gamma _{+}} p_{ij}^{*}(\varvec{x},\varvec{\xi },t) \Delta u_{j}(\varvec{x},t)\ d\Gamma (\varvec{x}) \\&\quad =\int _{\Gamma _{c}}u_{ij}^{*}(\varvec{x},\varvec{\xi },t) p_{j}(\varvec{x},t)\ d\Gamma \end{aligned}$$

32 $$\begin{aligned}&p_{j}(\varvec{\xi },t)+N_{r}\int _{\Gamma _{c}}s_{rij}^{*}(\varvec{x},\varvec{\xi },t) u_{j}(\varvec{x},t)\ d\Gamma (\varvec{x}) + N_{r}\int _{\Gamma _{+}}s_{rij}^{*}(\varvec{x},\varvec{\xi },t) \Delta u_{j}(\varvec{x},t)\ d\Gamma (\varvec{x}) \\&\quad =N_{r}\int _{\Gamma _{c}}d_{rij}^{*}(\varvec{x},\varvec{\xi },t) p_{j}(\varvec{x},t)\ d\Gamma (\varvec{x}) \end{aligned}$$

In this latter equation, the free term has been set to unity due to the additional singularity arising from the coincidence of the two crack surfaces. The inconvenience of this approach is that the BEM formulation will now involve integrals including both strong singularities which require special treatment. Numerous hypersingular approaches have been developed, in particular to anisotropic materials under static [90, 91, 150, 282] and time-harmonic [6, 93, 94, 226, 232, 283, 294] loadings. The use of a hypersingular formulation does not limit at all the crack shape, being valid for curved and branched cracks, for example. However, it is commonplace to make use of discontinuous boundary elements to ensure that all collocation points lie on the smooth surface within the body of an element; this is required to satisfy the Hölder continuity requirement of the hypersingular BIE.

As stated previously, the Stress Intensity Factors (SIF) are the measure of the stress amplification at the crack tip. They are used extensively when estimating the structural life in a number of applications, from civil engineering structures to aerospace devices. Therefore, a precise calculation of this parameter is essential. The principal difficulty, faced throughout the development of BEM and FEM approaches for modelling LEFM problems, is the use of these discrete techniques to capture the singular stress solution. Traditional finite element piecewise polynomial shape functions are ineffective. We now describe some common approaches to obtain the SIFs:

1. Quarter-point: Developed by Henshell and Shaw [119] and Barsoum [19] for finite elements, it consists in moving the mid-side node of a quadratic boundary element from the centre to 1/4 of the element length from the crack tip. It was shown that the mapping between the element in real space and in the space of the intrinsic coordinates automatically captures the asymptotic displacement behaviour of $1/\sqrt{r}$ present in the vicinity of the crack tip (refer to [231] for further explanations).
2. J-integral: Proposed by Rice [224], a path independent integral (assuming a non-curved crack) is used to evaluate the energy release rate due to the presence of the crack,
  33 $$J = \int _{\Gamma _j} \left( W n_1 - t_i \frac{\partial u_i}{\partial x_1} \right) d\Gamma$$
  where $n_1$ is the component of the outward unit normal vector in the $x_1$ direction, $u_i$ are the displacement and $t_i$ are the tractions. The term $W = \frac{1}{2}\sigma _{ij}\varepsilon _{ij}$ is the strain energy density.
3. Interaction integral: the J-integral can be decomposed into 3 parts [110, 176]
  34 $$J = J^{(1)} + J^{(2)} + M^{(1,2)}$$
  where $J^{(1)}$ is the J-integral of the so-called principal state, which represents the energy release rate of the material; $J^{(2)}$ is the J-integral of the auxiliary state, which depends on the displacements around the crack tip; $M^{(1,2)}$ is the interaction integral containing terms of the principal and auxiliary state, and is defined as
  35 $$M^{(1,2)}=\int _{A}(\sigma _{ij}^{(1)}u_{i,1}^{(2)}+\sigma _{ij}^{(2)}u_{i,1}^{(1)}-W^{(1,2)}\delta _{1j})q_{,j}\ dA$$
  where *A* is the area inside the contour $\Gamma _j$ surrounding the crack tip, and $W^{(1,2)}$ is given as
  36 $$W^{(1,2)}=\frac{1}{2}(\sigma _{ij}^{(1)}\varepsilon _{ij}^{(2)}+\sigma _{ij}^{(2)}\varepsilon _{ij}^{(1)})$$
  Let us remark that the indices (1) and (2) correspond to the principal and auxiliary states, respectively.

The quarter-point approach allows a direct extrapolation of the SIF by using the crack opening displacement. The J-integral is more cumbersome numerically since the displacements and tractions at the closed path integral are part of the BEM domain and have to be evaluated first; however it is more accurate than the direct extrapolation.

Chen [47] has analysed mixed mode SIFs of anisotropic cracks in rocks with a definition of the J-integral for anisotropic materials and the relative displacements at the crack tip. In Ke et al. [133], the authors have suggested a methodology to obtain the fracture toughness of anisotropic rocks through experimental measurements of the elastic parameters and further comparison with a BEM code. In another work, Ke et al. [132] have proposed a crack propagation model for transversely isotropic rocks. Let us remark that all the previously mentioned works have used the Lekhnitskii formalism [145] in order to model the anisotropy of the material. The Lekhnitskii formalism is a polynomial analogy form of the matricial Stroh formalism.

Crack propagation problems have also been studied under the BEM framework. Portela et al. [214] used the maximum stress criterion as crack growth criteria in a dual BEM. Quasi-static 3D crack growth is analysed in [169].

Cohesive models have also been developed with the BEM: Oliveira and Leonel [195, 196] have proposed a cohesive crack growth model, where the zone ahead of the crack tip is modelled as a fictitious crack model. This formulation gives rise to a volume integral, which must be regularised. The cohesive stresses are dependent on the crack tip opening displacement.

Yang and Ravi-Chandar [286] have proposed a cohesive model where the single-domain dual integral equations are used as an artifice to avoid the mathematical degeneration of the formulation imposed by the crack. In this case, the domain is divided in two sub-domains, where the crack is in the fictional domain division. Moreover, the cohesive zone is modelled as an elastic spring connecting both crack faces. Normal and tangential crack tip opening displacements are considered, and the crack growth is obtained from successive iterations of the non-linear system of equations, where the stiffness of the cohesive zone is taken into account.

Saleh and Aliabadi [233–235] and Aliabadi et al. [7] have studied the crack propagation problem in concrete using a fictitious crack tip zone. The cohesive zone is modelled with additional boundary elements at the fictitious crack tip that satisfy a softening cohesive law. A major drawback of this methodology is that the crack growth path has to be known a priori.

#### Fast Multipole Method (FMM)

The linear system formed in the BEM framework is much smaller than its equivalent with FEM formulation. However, the resulting matrix is full, not sparse like the FEM stiffness matrix, and this considerably increases the computational time required to solve a large problems. In 1985, Rokhlin [228] developed a method to reduce the complexity of solving the system of equations to $\mathcal {O}(n)$ instead of $\mathcal {O}(n^ 3)$, where *n* is the number of unknowns. This technique was named the Fast Multipole Method (FMM), and generally involves using an iterative solver (such as GMRES [230]) to solve the linear system

37 $${\mathbf {Ax}} = {\mathbf {b}}$$

which comes from the discretisation of Eqs. (31) and (32).

The Green’s functions in the BIEs can be expanded as follows

38 $$u_{ij}^{*}(\varvec{x},\varvec{\xi },t) = \sum _i u_{ij}^{*\xi }(\varvec{x}_e, \varvec{\xi },t) u_{ij}^{*x}(\varvec{x}_e,\varvec{\xi },t)$$

where ${\mathbf {x}}_e$ is an expansion point near ${\mathbf {x}}$ obtained through Taylor series expansion, for instance. The original integral containing $u_{ij}^*$ can be rewritten as

39 $$\int _{\Gamma _{a}}u_{ij}^{*}(\varvec{x},\varvec{\xi },t) p_{j}(\varvec{x},t) \ d\Gamma = u_{ij}^{*\xi }(\varvec{x}_e, \varvec{\xi },t) \int _{\Gamma _a} u_{ij}^{*x}(\varvec{x}_e,\varvec{\xi },t) p_{j}(\varvec{x},t)\ d\Gamma$$

where $\Gamma _a$ is a boundary away from $\varvec{\xi }$. This change allows the collocation point $\varvec{\xi }$ to be independent of the observation point ${\mathbf {x}}$ due to the introduction of a new point ${\mathbf {x}}_e$. Equation (39) has to be evaluated only once for different collocation points.

**Fig. 7:**
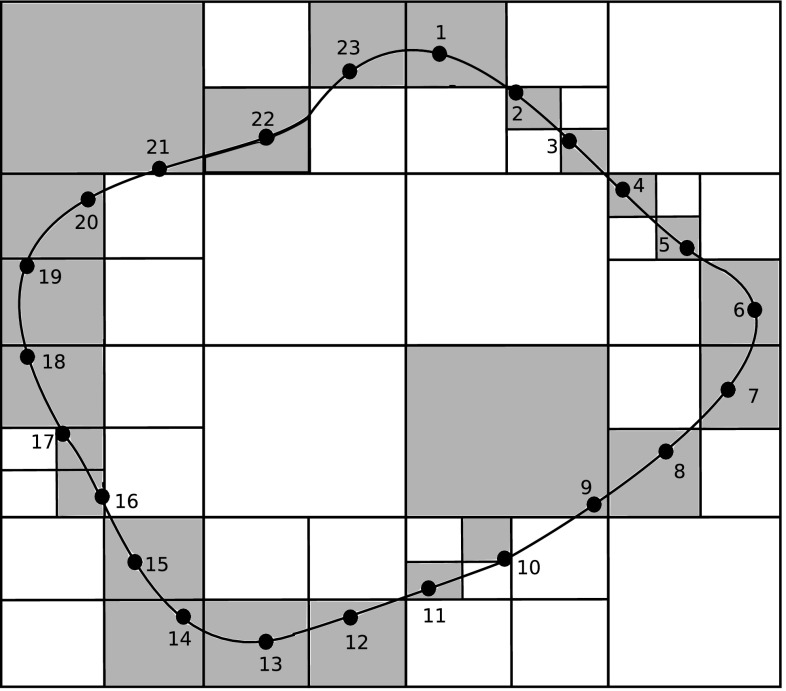
Hierarchical tree structure

**Fig. 8:**
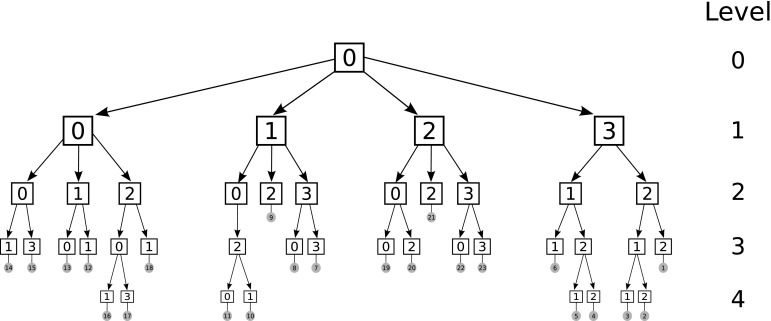
Hierarchical quad-tree structure

The FMM applied in BEM can be described by the following steps [150]:

1. Discretise the boundary $\Gamma$;
2. Determine a tree structure of the elements. For example, in a 2D domain, define a square containing the entire boundary and call this square the cell of level 0. Then, divide the square into 4 equal cells and call them level 1. Repeat until each cell contains a predetermined number of elements (in Fig. 7, each cell has one element). Cells with no children cell are called leafs. For 3D cases, the same principle applies using cubic cells instead of square cells;
3. Compute the moments on all cells for all levels $l \ge 2$ and trace the tree structure (shown in Fig. 8). The moment is the term from Eq. (39) that is independent from the collocation point. The moment of parent cells is calculated from the summation of the moments of its 4 children cells;
4. Compute the local expansion coefficients on all cells starting from level 2 and tracing the tree structure downward to all leaves. The local expansion of the cell *C* is the sum of the contributions from the cell in the interaction list of the cell and the far cells. The interaction list is composed by all the cells from the level *l* that do not share any common vertices with other cells at the same level, but their parent cells do share at least one common vertex at level $l-1$. Cells are said to be far cells of *C* if their parent cells are not adjacent to the parent cell of *C*;
5. Compute the integrals from element in leaf cell *C* and its adjacent cells as in standard BEM. The cells in the interaction list and the far cells are calculated using the local expansion;
6. Obtain the solution of ${\mathbf {Ax}}={\mathbf {b}}$. The iterative solver updates the unknown solution of ${\mathbf {x}}$ and goes to step 3 to evaluate the next matrix vector product ${\mathbf {Ax}}$ until the solution converges within a given tolerance.

The FMM has been used in 3D fracture mechanics problems as can be seen in [192, 290], and some recent works on GPU can be found in [101, 108, 278]. The FMM is largely detailed in [149].

#### Adaptive Cross Approximation (ACA)

The Adaptive Cross Approximation (ACA) approach uses a different technique in order to reduce the complexity of the BEM with respect to the storage and operations. ACA uses the concept of hierarchical matrices introduced by Hackbusch [107], where a geometrically motivated partitioning into sub-blocks takes place, and each sub-block is classified as either admissible or inadmissible according to the separation of the node clusters within them.

The main idea is that admissible blocks are approximated by low-rank approximants formed as a series of outer products of row and column vectors, greatly accelerating the evaluation of the matrix vector product that lies within each iteration of an iterative solver. While the FMM deals with the analytical decomposition of the integral kernels, ACA can evaluate only some original matrix entries, or use a full pivoted form where all terms of matrix are calculated, to get an almost optimal approximation. The approximation of matrix ${\mathbf {A}} \in C^{t\times S}$ is given by

40 $${\mathbf {A}} \approx S_k = {\mathbf {UV}}^t,\quad {\text { where }}\quad {\mathbf {U}} \in C^{t\times k}\quad {\text { and }} \quad{\mathbf {V}} \in C^{s\times k}$$

where *k* is a low-rank compared to *t* and *s*. It is important to remark that the low-rank representation can only be found when the generating kernel function in the computational domain of ${\mathbf {A}}$ is asymptotically smooth. It has been shown in [20] that elliptic operators with constant coefficients have this property.

In hierarchical matrices, the near and far fields have to be separated. The index sets *I* for row and *J* for columns so that elements far away will have indices with a large offset.

By means of a distance based hierarchical subdivision of *I* and *J* cluster trees $T_I$ and $T_J$ are created. In each step of this procedure, a new level of son clusters is inserted into the cluster trees. A son cluster is not further subdivided and is said to be a leaf if its size reaches a prescribed minimal size $b_{min}$. Usually one of two different approaches is considered. First, a subdivision based on bounding boxes splits the domain into axis-parallel boxes which contain the son clusters. Alternatively, a subdivision based on principal component analysis splits the domain into well-balanced son clusters leading to a minimal cluster tree depth.

Now, the hierarchical ($\mathcal {H}$)-matrix structure is defined by the block cluster tree $T_{IJ}=T_I \times T_J$ using the following admissibility criterion: $min(diam(t), diam(s))\le \eta dist(t,s)$, with the clusters $t \subset T_I, s \subset T_J$, and the admissibility parameter $0 \,<\, \eta \,< \,1$. The diameter of the clusters *t* and *s*, and their distance, are obtained as

41 $$diam(t)= \underset{i_1,i_2 \in\ t}{max} |\varvec{\xi }_{i_1}-\varvec{\xi }_{i_2}|$$

42 $$diam(s)= \underset{j_1,j_2 \in\ s}{max} |\varvec{x}_{j_1}-\varvec{x}_{j_2}|$$

43 $$dist(t,s)= \underset{i\in\ t,\ j\in\ s}{|\varvec{\xi }_i - {\mathbf {x}}_j |}$$

A block *b* is said to be admissible if it satisfies this admissibility criterion. Otherwise, the admissibility is recursively verified for each son cluster, until the block becomes admissible or reaches the minimum size.

Finally, the ACA method idea is to split the matrix ${\mathbf {A}} \in C^{t\times s}$ into ${\mathbf {A}} = {\mathbf {S}}_k + {\mathbf {R}}_k$, where ${\mathbf {S}}_k$ is the rank *k* approximation and ${\mathbf {R}}_k$ is the residuum which has to be minimised. We now present the ACA method itself:

1. Define $k=0$ where ${\mathbf {S}}_0={\mathbf {0}}$ and ${\mathbf {R}}_0={\mathbf {A}}$ and the first scalar pivot to be found is $\gamma _1 = (R_0)^{-1}_{ij}$, and *i*, *j* are the row and column indices of the actual approximation step;
2. For each step $\upsilon$, obtain
  44 $$v_{\upsilon +1}= \gamma _{\upsilon +1} (R_\upsilon )_i$$
  45 $$u_{\upsilon +1}= (R_\upsilon )_j$$
  46 $${\mathbf {R}}_{\upsilon +1}= {\mathbf {R}}_\upsilon - {\mathbf {u}}_{\upsilon +1} {\mathbf {v}}_{\upsilon +1}^t$$
  47 $${\mathbf {S}}_{\upsilon +1}= {\mathbf {S}}_\upsilon + {\mathbf {u}}_{\upsilon +1} {\mathbf {v}}_{\upsilon +1}^t$$
  where the operators $()_i$ and $()_j$ indicate the *i*-th row and the *j*-th column vectors, respectively;
3. The next pivot $\gamma _{\upsilon +1}$ is chosen to be the largest entry in modulus of the row $(R_\upsilon )_i$ or the column $(R_\upsilon )_j$
4. The approximation stops when the following criterion holds
  48 $$||u_{\upsilon +1}||_F\ ||v_{\upsilon +1}||_F < \varepsilon\ ||{\mathbf {S}}_{\upsilon +1}||_F$$

The main advantage comparing to the FMM method is that ACA can be implemented directly into an existing BEM code. Moreover, due to its inherently parallel data structure, parallel programming can be readily implemented, increasing the computational efficiency. However, the original matrix ${\mathbf {A}}$ will not be entirely recovered.

Note that it is not necessary to build the whole matrix beforehand. The respective matrix entries can be computed on demand [20]. Working on the matrix entries has the advantage that the rank of the approximation can be chosen adaptively while kernel approximation requires an a priori choice.

A few recent works on ACA implementation can be found in [81, 99]. Use of the method for problems in 3D elasticity can be found in [28, 158] and the application of ACA in crack problems was shown for the first time in [137].

### Enriched Formulations

#### eXtended Finite Element Method (X-FEM)

The motivation that lay behind the development of X-FEM was to eliminate some of the deficiencies of standard FEM for crack modelling, most importantly the requirement for highly refined meshing around the crack tips and the mandatory remeshing for crack growth problems. The partition of unity [15] is a general approach that allows the enrichment of finite element approximation spaces so that the FEM has better convergence properties. In X-FEM, the partition of unity method allows element enrichment such that degrees of freedom (dofs) are added to represent discontinuous behaviour. In this framework, the mesh is independent from the discontinuities, so that cracks may now pass through elements rather than being constrained to propagate along elment edges. This gives the FEM much more flexibiility to model crack growth without remeshing.

Two types of enrichment function are applied in the X-FEM: the Heaviside enrichment function, responsible for characterising the displacement discontinuity across the crack surfaces, and a set of crack tip enrichment functions (CTEFs), responsible for capturing the displacements asymptotically around the crack tip. This latter presents complex behaviour, varying for different constitutive laws (see [12, 79, 193], for some different CTEF). In this sense, it is similar to the FS, necessary in BEM formulations.

The displacement approximation ${\mathbf {u}}^{h}({\mathbf {x}})$ with the partition of unity can be stated as [176]

49 $${\mathbf {u}}^{h}({\mathbf {x}})=\sum _{i\in {\mathcal{ N}}}N_{i}({\mathbf {x}}){\mathbf {u}}_{i}+\sum _{j\in {\mathcal {N}}^{H}}N_{j}({\mathbf {x}})H({\mathbf {x}}){\mathbf {a}}_{j}+ \sum _{k\in {\mathcal {N}}^{CT}}N_{k}({\mathbf {x}})\sum _{\alpha }F_{\alpha }({\mathbf {x}}){\mathbf {b}}_{k}^{\alpha }$$

where $N_{i}$ is the standard finite element shape function associated with node $i, {\mathbf {u}}_{i}$ is the vector of nodal dofs for classical finite elements, and ${\mathbf {a}}_{j}$ and ${\mathbf {b}}_{k}^{\alpha }$ are the added set of degrees of freedom that are associated with enriched basis functions, associated with the Heaviside function $H({\mathbf {x}})$ and the CTEF $F_{\alpha }({\mathbf {x}})$, respectively. $\mathcal N$ is the set of all nodes, $\mathcal N^{H}$ is the set of all nodes lying on crack surfaces, and $\mathcal N^{CT}$ is the set of all nodes belonging to elements touching a crack tip.

Since the CTEFs describe the displacements at the crack tip zone through the addition of several dofs, the stress concentration around the crack tip can be found more accurately with a significantly coarser mesh compared to the mesh used with standard FEM in a similar problem.

The presence of blending elements, which do not contain the crack but contain enriched nodes, is also important and has to be considered. These elements were analysed by Chessa et al. [48], and some studies have improved the convergence of blending elements (see [84], for instance). The X-FEM convergence rate can also be increased through the use of geometrical enrichment [142], where a number of elements around the crack tip receive the CTEF instead of a single element (this latter named topological enrichment).

**Fig. 9:**
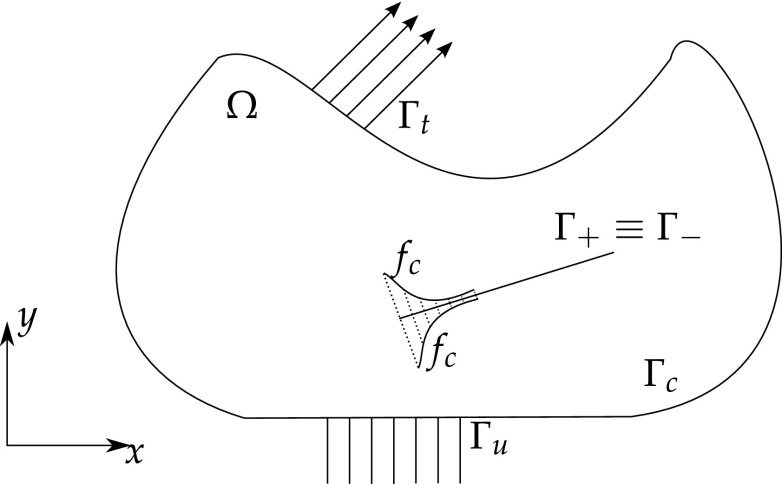
Elastic body with a cohesive crack

Figure 9 illustrates an arbitrary elastic body with a cohesive crack. The governing equations for a cohesive crack model are given by [178]

50 $$\int _\Omega \varvec{\sigma }. \delta \varvec{\epsilon }\ d\Omega = \int _\Omega {\mathbf {f}}^b. \delta {\mathbf {u}}\ d\Omega + \int _{\Gamma _t} \alpha {\mathbf {f}}^t.\delta {\mathbf {u}}\ d\Gamma + \int _{\Gamma _+ \cup \ \Gamma_-} {\mathbf {f}}^c.(\delta {\mathbf {u}}^+-\delta {\mathbf {u}}^-)\ d\Gamma$$

where $\Omega$ is the domain, ${\mathbf {f}}^b$ is the body force vector, ${\mathbf {f}}^t$ is the external traction vector, $\varvec{\sigma }$ is the stress tensor, $\alpha$ is the load factor which controls the load increments, $\mathbf f^c$ is the traction along the cohesive zone, and is a function of the crack opening $\Delta u$.

The discretisation of Eq. (50) yields

51 $${\mathbf {Ku}} = {\mathbf {f}}^{ext} + {\mathbf {f}}^{cohe}$$

with

52 $${\mathbf {K}}= \int _\Omega {\mathbf {B}}^t {\mathbf {C}} {\mathbf {B}}\ d\Omega$$

53 $${\mathbf {f}}^{ext}= \alpha \int _{\Gamma _c} N_i \overline{t}\ d\Gamma + \int _\Omega N_i {\mathbf {b}}\ d\Omega$$

54 $$\mathbf f^{cohe}= -2\int _{\Gamma _+ \cup \ \Gamma_-} N_i {\mathbf {T}}^c(\Delta u)\ d\Gamma$$

where ${\mathbf {B}}$ is the finite element strain-displacement matrix, ${\mathbf {b}}$ is the vector containing the body forces and ${\mathbf {T}}^c(\Delta u)$ is the cohesive softening law relating the crack surface normal traction ${\mathbf {f}}^c$ to the crack opening $\Delta u$.

X-FEM has been widely used with cohesive models in the last few years. Some authors [45, 51, 175] have used a typical X-FEM formulation to model the cohesive crack, i.e., a Heaviside enrichment function is used to model the jump between the crack surfaces and a crack tip enrichment function is used to model the asymptotic behaviour at the crack tip.

Xiao and Karihaloo [284] have obtained the asymptotic displacement at the cohesive zone for isotropic materials based on the Williams expansion. The authors considered only the case where the crack is traction free and the crack is subject only to mode I. The obtained enrichment functions are

55 $$u_1^{tip}= \frac{r^{3/2}}{2\mu }a_{11}\left[ \left( \kappa +\frac{1}{2}\right) \cos {\frac{3}{2}\theta }-\frac{3}{2}\cos {\frac{1}{2}\theta }\right]$$

56 $$u_2^{tip}= \frac{r^{3/2}}{2\mu }a_{11}\left[ \left( \kappa -\frac{1}{2}\right) \sin {\frac{3}{2}\theta }-\frac{3}{2}\sin {\frac{1}{2}\theta }\right]$$

where $\kappa$ is the Kolosov constant (for details refer to [284]), $\theta$ is the crack orientation with respect to the $x_1$ axis, $a_{11}$ is a real constant and comes from the Williams expansion. In this case, Eq. (57) receives a new crack tip enrichment term, as in the X-FEM formulation for linear elastic fracture mechanics (see [110, 175]). Zamani et al. [291] uses higher-order terms of the crack tip asymptotic field to obtain an enrichment function based on the Williams expansion.

This approach has provided good results for isotropic materials, but it may not be the same for anisotropic materials. An alternative approach is to model the crack with Heaviside elements only [139, 180, 262, 302]. Since there is no discontinuity at the crack tip, there are no SIFs at the crack tip, and therefore no crack tip enrichment function is required. The displacement field ${\mathbf {u}}({\mathbf {x}})$ is given by

57 $${\mathbf {u}}({\mathbf {x}}) = \sum _{i \in {\mathcal{ N}}} N_i({\mathbf {x}}){\mathbf {u}}_i + \sum _{k\in {\mathcal{ N}}^{H}} N_k({\mathbf {x}}) H({\mathbf {x}}){\mathbf {a}}_j$$

where $N_i$ is the standard finite element shape function associated to node *i* and ${\mathbf {a}}_j$ is the additional set of degrees of freedom associated with the Heaviside enrichment function *H*, defined as $+1$ if it is evaluated above the crack or $-1$ if below the crack. The sets $\mathcal N$ and $\mathcal N^H$ denote the standard and enriched nodes, respectively.

The crack growth is modelled considering some rules, for example, if the level of stress at the crack tip is above the material tensile strength [178, 262].

In [139], a 2D cohesive model for an isotropic material was presented, where both fluid and porous material interact. The pressure inside a crack is also modelled. The Heaviside enrichment function is employed, as well as a pressure enrichment function, which allows the continuity of steep gradients without enforcing this condition. The crack propagation criteria depends on the stress state at the crack tip. The fluid behaviour can retard crack initiation and propagation. A local change of the flow can be seen immediately after crack propagation. The deformation around the crack causes fluid to flow mostly from the crack itself since the crack permeability is much higher than the medium permeability. This flow from the crack to the crack tip causes closing of the crack. However, a delamination test has shown that if the stiffness and permeability are high, the fluid does not influence crack growth.

More methods for crack propagation in X-FEM can be found in [151, 167, 182, 183, 225] for brittle fracture and [168, 179, 291] for cohesive cracks.

#### Enriched BEM

The extended boundary element method (X-BEM) was first proposed by Simpson and Trevelyan [251] for fracture mechanics problems in isotropic materials. The main idea is to model the asymptotic behaviour of the displacements around the crack tips by introducing new degrees of freedom. The displacements ${\mathbf {u}}^h({\mathbf {x}})$ are thus redefined as

58 $${\mathbf {u}}^{h}({\mathbf {x}})=\sum _{i\in {\mathcal{ N}}}N_{i}({\mathbf {x}}){\mathbf {u}}_{i}+ \sum _{k\in {\mathcal{ N}}^{CT}}N_{k}({\mathbf {x}})\sum _{\alpha }F_{\alpha }({\mathbf {x}}){\mathbf {a}}_{k}^{\alpha }$$

where $\mathcal N$ and $\mathcal N^{CT}$ are the sets with non-enriched and enriched nodes, respectively, $N_{i}$ is the standard Lagrangian shape function associated with node $i, {\mathbf {u}}_{i}$ is the vector of nodal degrees of freedom, and ${\mathbf {a}}_{k}^{\alpha }$ represents the enriched basis functions which capture the asymptotic behaviour around the crack tips. In elastic materials, ${\mathbf {a}}_{k}^{\alpha }$ is an 8-component vector for two-dimensional problems, since only two nodal variables ($u_{1}$, $u_{2}$) and four enrichment functions are needed to describe all the possible deformation states in the vicinity of the crack tip [110].

Hattori et al. [110] used the following anisotropic enrichment functions initially developed for the X-FEM

59 $$F_{l}(r,\theta )=\sqrt{r}\left( \begin{array}{c} {\mathfrak {R}}\{A_{11}B^{-1}_{11}\beta _{1}+A_{12}B^{-1}_{21}\beta _{2}\}\\ {\mathfrak {R}}\{A_{11}B^{-1}_{12}\beta _{1}+A_{12}B^{-1}_{22} \beta _{2}\} \\ {\mathfrak {R}}\{A_{21}B^{-1}_{11}\beta _{1}+A_{22}B^{-1}_{21}\beta _{2}\} \\ {\mathfrak {R}}\{A_{21}B^{-1}_{12}\beta _{1}+A_{22}B^{-1}_{22} \beta _{2}\} \\ \end{array}\right)$$

where $\beta _i = \sqrt{\cos {\theta } + \mu _i \sin {\theta }}, r$ is the distance between the crack tip and an arbitrary position, $\theta$ is the orientation measured from a coordinate system centred at the crack tip, and ${\mathbf {A}}, {\mathbf {B}}$ and $\varvec{\mu }$ are obtained from the following eigenvalue problem60(no sum on m) with

61 $${\mathbf {Z}}:= \mathbf {C}_{1ij1}; \quad {\mathbf {M}}:= \mathbf {C}_{2ij1}; \quad {\mathbf {L}}:= \mathbf {C}_{2ij2}$$

Let us emphasise that the anisotropic enrichment functions can also be used for isotropic materials, since this is a degenerated case from anisotropic materials. For more details please refer to reference [110].

An enriched anisotropic dual BEM formulation using the above enrichment functions [111] for anisotropic materials is similar to the one used by Simpson and Trevelyan [251] for isotropic materials. The extended DBIE and the TBIE can be restated as

62 $$\begin{aligned}&c_{ij}(\varvec{\xi })u_{j}(\varvec{\xi })+\int _{\Gamma } p_{ij}^{*}(\varvec{x},\varvec{\xi })u_{j}(\varvec{x})\ d\Gamma (\varvec{x}) + \int _{\Gamma _c}p_{ij}^*(\varvec{x},\varvec{\xi }) F_\alpha (\varvec{x}) {\mathbf {a}}_k^\alpha\ d\Gamma \nonumber \\&\quad =\int _{\Gamma }u_{ij}^{*}(\varvec{x}, \varvec{\xi })p_{j}(\varvec{x})\ d\Gamma (\varvec{x}) \end{aligned}$$

63 $$\begin{aligned}&c_{ij}(\varvec{\xi })p_{j}(\varvec{\xi }) +N_{r}\int _{\Gamma }s_{rij}^{*}(\varvec{x},\varvec{\xi })u_{j}(\varvec{x} )\ d\Gamma (\varvec{x}) + N_r \int _{\Gamma _c}s_{rij}^*(\varvec{x},\varvec{\xi }) F_\alpha (\varvec{x}) {\mathbf {a}}_k^\alpha\ d\Gamma \nonumber \\&\quad =N_{r}\int _{\Gamma }d_{rij}^{*}(\varvec{x},\varvec{\xi }) p_{j}(\varvec{x})\ d\Gamma (\varvec{x}) \end{aligned}$$

where $\Gamma _c = \Gamma _+ \cup \Gamma _-$ stands for the crack surfaces $\Gamma _+$ and $\Gamma _-$. Only the element containing the crack tip receives the enrichment function. Strongly singular and hypersingular terms arise from the integration of the $p_{ij}^*$, $d_{rij}^*$ and $s_{rij}^*$ kernels and they may be regularised in the same way as shown in [92].

### Meshless Method

Meshless (or meshfree) methods have been the subject of considerable interest in recent years as alternatives to Finite Element (FE) methods for solid mechanics problems. As the name suggests the main advantage of these methods is their (varying) lack of reliance on a division of the problem domain into a mesh of elements, thus removing issues associated with mesh generation and remeshing (perhaps required following large deformations which would lead to distorted and hence inaccurate elements). However, meshless methods tend to be more computationally expensive largely as a result of the lack of easily consultable connectivity information provided by a mesh, but also because there is greater complexity in the formation of shape functions. The most popular meshless methods for solid mechanics are the Element-free Galerkin method [24] and the Meshless Local Petrov–Galerkin method [14]. The key difference in both of these methods compared to FE methods is the use of shape functions based on a moving least squares (MLS) approximation [77]. Taking the Element-free Galerkin method as an example, the displacement approximation $u^h$ at location ${\mathbf {x}}$ is constructed as

64 $$u^h({\mathbf {x}})=\sum _{i\in {\mathcal{N}}}N_i({\mathbf {x}})u_i=\mathbf {N} {\mathbf {u}}$$

where $N_i$ are shape functions based on the MLS approximation (explained below), $u_i$ are nodal values and $\mathcal {N}$ is the set of nodes in support at location ${\mathbf {x}}$, supports being defined using weighting functions centred at nodes. To build the shape functions we choose a polynomial basis, which can be of any order but low orders are usually used, e.g. a quadratic basis in 1D ${\mathbf {p}}({\mathbf {x}})^T = \left\{ 1, x, x^2\right\}$ or in 2D ${\mathbf {p}}({\mathbf {x}})^T = \left\{ 1, x, y, x^2, xy, y^2\right\}$. At any location ${\mathbf {x}}$ we define the matrix ${\mathbf {P}}$ whose rows are the valued basis vectors ${\mathbf {p}}^T$ for the nodes in support at ${\mathbf {x}}$. A least squares minimisation procedure applied to the approximation at node locations and the nodal values then leads to the shape functions as

65 $${\mathbf {N}}={\mathbf {p}}({\mathbf {x}})^T{\mathbf {A}}({\mathbf {x}})^{-1}{\mathbf {B}}{{\mathbf {x}}}$$

where

66 $${\mathbf {A}} = {\mathbf {P}}^T {\mathbf {W}} {\mathbf {P}}, \quad {\mathbf {B}} = {\mathbf {P}}^T{\mathbf {W}}.$$

${\mathbf {W}}$ is a diagonal matrix of values of node-centred weight functions at location ${\mathbf {x}}$, which may be splines or exponential functions. Carrying this out in 2D or 2D is simply done via tensor products of the 1D case. Key points of difference as compared to FE methods should be clear, i.e. the shape function formation requires the inversion of a matrix, albeit a small matrix (dimension same size as the number of terms in the basis), the choice of nodal support size is crucial but not easy to define and the use of an MLS approximation contrasts with the interpolation used in FE methods and has the knock-on effect of making the imposition of essential boundary conditions more complicated. Overviews of the various meshless methods for solid mechanics can be found in a number of references [23, 85, 147, 191].

#### Meshless Methods for Fracture

Ever since their initial development in the 1990s meshless methods have been applied to crack modelling [22, 24, 197], to dynamic fracture [26] and crack propagation [25]. The key advantage of meshless methods over standard FE methods for fracture is removal of the need to remesh during crack propagation. Another positive feature of meshless methods is that smooth stress results can be obtained for high stress gradients around crack tips [37] thus requiring less effort in postprocessing compared with the X-FEM. As with all numerical methods applied to fracture we have to find ways of dealing with the stress singularities at the crack tips and the discontinuities introduced by the crack surfaces. The former can be dealt in meshless methods by enriching the approximation space just as is done in X-FEM and other enriched methods, e.g. [27], based on the the partition of unity (PU) concept [16, 166] where the jump discontinuity is included in the displacement approximation exactly as already laid out for X-FEM above in Eq. (49). “Extrinsic” techniques like this have more recently been developed into meshless “cracked particle” methods in a number of references [37, 219, 220, 303]. Extrinsic enrichment like this can however lead to an ill-conditioned global stiffness matrix [21] as is the case with many other PU methods, due to the additional unknowns at nodes which do not correspond to the physical degrees of freedom [44]. The cracked particle methods are examples of smeared approaches to modelling cracks, i.e. the exact crack face/surface geometry is approximated, but this clashes with the requirement for an accurate description of the crack geometry since it governs the accuracy of field solution, and hence the crack growth magnitude and direction. Extrinsic approaches which attempt to improve on this have used piecewise triangular facets [37, 64] which however suffer from discontinuous crack paths and requires user input to “repair” the mesh of facets.

**Fig. 10:**
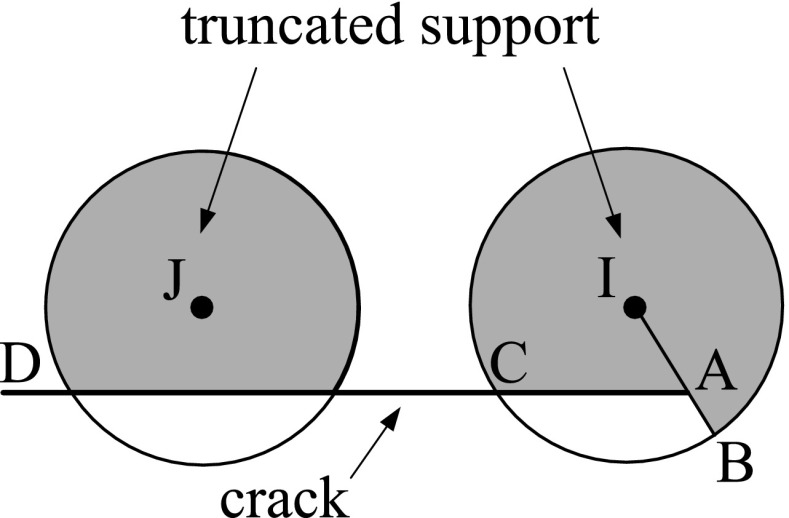
The visibility criterion (from [300])

**Fig. 11:**
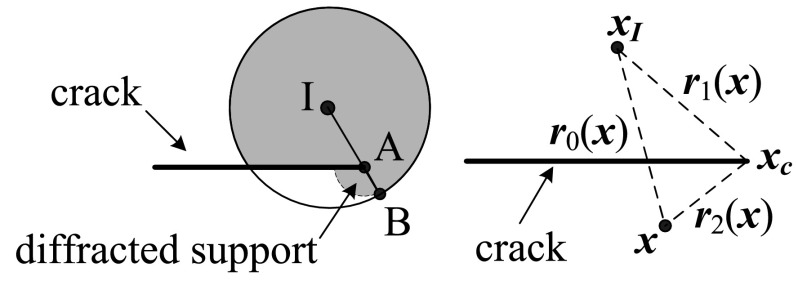
The diffraction method (from [300])

Greater promise lies in the use of a level set description of crack geometry combined with a meshless method [65, 98, 300, 301] and an intrinsic rather than extrinsic model of the discontinuity of a crack. Using an intrinsic method in the EFGM there is also no problem of ill-conditioning in the stiffness matrix. Here the displacement jump can be introduced simply by modifying the nodal support via the weight function. A simple way to do this is directly to truncate the nodal support at a crack face. This is the visibility criterion, as shown in Fig. 10. The support of a node is restricted to areas of the domain visible from the node with the crack faces acting as an opaque barrier. If a line between a node and the point of interest intersects a crack, and if the crack tip is inside the support of that node, the node will have no influence on that point, i.e. $r_I$ between that point and the node is modified to infinity. (The visibility criterion corresponds to the use of the Heaviside function in the enriched trial functions used in X-FEM). An alternative to the visibility criterion is the diffraction method which works slightly differently as shown in Fig. 11.

The visibility criterion is simpler to implement, especially for 3D problems, but leads to spurious crack extension (thus impairing accuracy) while the diffraction method has no spurious crack extension problem but its implementation leads to high computational complexity especially in 3D or with multiple cracks.

**Fig. 12:**
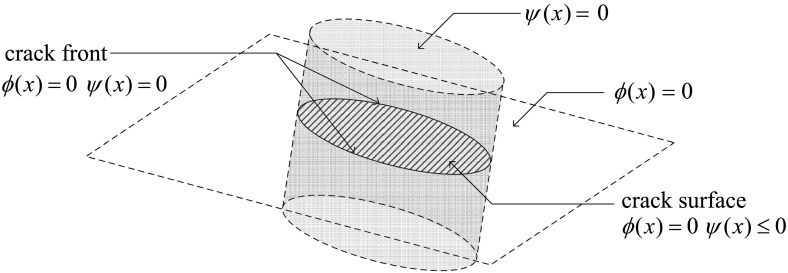
The level sets description of a crack surface in 3D (from [301])

Level sets offer a means accurately to represent crack surfaces and also to track surfaces as crack fronts propagate. The level set method (LSM) is a computational geometry technique for tracking interfaces applicable to many areas in science and engineering [243]. The LSM was first applied to crack modelling using the X-FEM in [98]. Instead of using an explicit representation of a crack, such as line segments in 2D and triangular facets in 3D, the LSM describes the surface implicitly by collecting points at the same distance to the crack into level sets. When the LSM is applied to fracture modelling, two orthogonal level sets, $\phi$ and $\psi$ are used: $\phi$ measures the distance normal to the crack and $\psi$ measures the distance tangential to the crack (see Fig. 12).

Hence we can fully define the geometry of the crack surface as

67 $$\begin{array}{ll} \phi ({\mathbf {x}}) = 0,\quad \psi ({\mathbf {x}}) \le 0 & \mathrm {crack\,surface}\\ \phi ({\mathbf {x}}) = 0,\quad \psi ({\mathbf {x}}) = 0 & \mathrm {crack\,front}. \end{array}$$

As the crack propagates, the level sets are updated to the new crack surface using the procedures in [98] and the corrected update function for $\phi$ in [65]. Recent work has led to the development of a fracture modelling method for 2D and 3D which uses intrinsic LS representations of cracks using a modified visibility criterion where the crack tips are tied to avoid the spurious propagation problem, and also incorporates enrichment to deal with the stress singularities [300, 301].

### Phase-Field

**Fig. 13:**
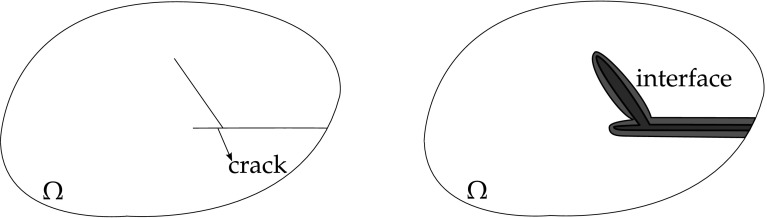
Phase-field domain

The development of the phase-field method provided an alternative formulation when dealing with different interface problems. A phase-field variable is introduced to consider the interface directly into the formulation. The phase-field formulation has been applied to different types of interface problems, including liquid-solid [13], liquid-solid-gas [165], electromagnetic wave propagation [260], analysis in crystal structures [1, 58] and more recently in medicine [266], to enumerate some of the applications. The method has been successfully applied to fracture mechanics problems, where the crack is therefore modelled as a different interface in the domain. Figure 13 shows an example of a domain where the damage state is given by the interface parameter.

The work of Francfort and Marigo [80] is the first in fracture mechanics to consider a variational formulation where a parameter assumes different values in order to capture the proper interface in the domain. An energy functional $E({\mathbf {u}},\Gamma )$, depending on the displacement field ${\mathbf {u}}$ and the crack surface $\Gamma$, is defined as [8, 80]

68 $$E({\mathbf {u}}, \Gamma ) = E_d({\mathbf {u}}) + E_s(\Gamma ) = \int _\Omega \psi _0(\varepsilon ({\mathbf {u}}))\ d\Omega + G_c\int _\Gamma \ ds$$

where $E_d({\mathbf {u}})$ represents the elastic energy of the body, $E_s(\Gamma )$ is the energy required to create the crack, considering Griffith’s theory, $\psi _0$ is the elastic energy density and $G_c$ is the material fracture toughness. The work is further extended by [39] which applied a regularised form in order to allow the numerical treatment of the energy functional. The regularised energy functional $E_\epsilon ({\mathbf {u}}, \Gamma )$ is given by

69 $$E_\epsilon ({\mathbf {u}},\Gamma ) = \int _\Omega (s^2+k_\epsilon )\psi _0(\varepsilon ({\mathbf {u}}))\ dx + G_c\int _\Omega \left( \frac{1}{4\epsilon }(1-s^2)+\epsilon |\nabla s|^2 \right) dx$$

where *s* is the phase-field variable, $s=0$ representing the undamaged state and $s=1$ standing for the fully broken/damaged state, with $0\le s \le 1; \epsilon > 0$ is a parameter designed to control the width of the transition zone set by the phase-field variable, and $k_\epsilon$ is a small term depending on $\epsilon$.

The solution of Eq. (69) is found through the minimisation of $E_\epsilon ({\mathbf {u}},\Gamma )$. To avoid the minimisation problem to be ill-posed, the small term $k_\epsilon$ has been added to the formulation. For more details see [39].

The phase-field formulation has been modified through the years to be more general, consider more cases of interface interaction and different types of loading conditions to the problem. The work of Amor et al. [9] has considered the compression into the formulation, avoiding the interpenetration between crack surfaces. The proposed idea consisted in separating the elastic energy density according to the deviatoric and volumetric contributions.

A different phase-field formulation was proposed by [172, 173], defined as a “thermically consistent” formulation. The regularised phase-field variable *d* is defined as 0 for the unbroken state and 1 for the fully broken state.

The stored energy $\psi _0(\varepsilon )$ of an undamaged solid is defined as [173]

70 $$\psi _0(\varepsilon ) = \psi _0^+(\varepsilon ) + \psi _0^-(\varepsilon )$$

where $\psi _0^+(\varepsilon )$ is the energy due to tension and $\psi _0^-(\varepsilon )$ is the energy due to compression. The positive and negative parts of the energy are given by the following decomposition of the strain tensor

71 $$\varepsilon = \sum _{a=1}^3\varepsilon _a {\mathbf {n}}_a \otimes {\mathbf {n}}_a$$

where $\epsilon _a$ and ${\mathbf {n}}_a$ are the principal strain and principal strain direction in the $x_a$-axis, respectively. The standard quadratic energy storage function of an isotropic undamaged material is given by

72 $$\psi _0(\epsilon ) = \frac{1}{2}\lambda (\varepsilon _1+\varepsilon _2+\varepsilon _3)^2 + \mu (\varepsilon _1^2+\varepsilon _2^2+\varepsilon _3^2)$$

with $\lambda > 0$ and $\mu > 0$ are elastic constants.

The phase-field model for fracture in elastic solids is given by

73 $$Div \left( (1-d)^2+k \right) \frac{\partial \psi _0^+(\varepsilon ({\mathbf {u}}))}{\partial \varepsilon } + \frac{\partial \psi _0^-(\varepsilon ({\mathbf {u}}))}{\partial \varepsilon } = 0$$

74 $$\frac{G_c}{l}(d-l^2 \Delta d)-\left( 2(1-d) \psi _0^+(\varepsilon ({\mathbf {u}})) + \varepsilon \langle \dot{d} \rangle _- \right) = 0$$

where *Div* represents the divergent, $\Delta d$ is the Laplacian of the phase-field, *l* is the width of the transition zone (where $0<d<1$), *k* is a small artificial residual stiffness to prevent the full-degradation of the energy at the fully damaged state $d=1$, $\langle x \rangle _- = (|x|-x)/2$ is a ramp function, $\dot{d}$ is the evolution of the phase-field parameter.

A downside of the phase-field formulation is that it can result in unrealistic solutions. An example analysed by [8] consists of the case when the principal strains are negative, which is not considered in the model of [9] for instance. Nevertheless, a strongly non-linear strain relation is used, which requires higher computational charges as compared to [9].

A history-field variable was introduced in [172] in order to overcome some implementations issues which arose in [173]. Since the $\psi _0^+$ term determines the phase-field variable, we have

75 $${\mathscr {T}}({\mathbf {x}}, t) = \max _{s \in [0,t]}\ \psi _0^+(\varepsilon ({\mathbf {x}},s))$$

Substituting Eq. (75) into (74) and applying a viscous regularisation, the evolution equation can be recast as

76 $$\frac{G_c}{l}(d-l^2 \Delta d) = 2(1-d){\mathscr {T}} + \eta \dot{d}$$

where $\eta >0$ is a viscous parameter.

The advantage of this new form is that the irreversibility of the crack phase-field evolution is put into a more general form, allowing loading/unloading conditions, besides allowing a better numerical treatment of the phase-field.

Crack branching effects are studied with phase-field in [117] for a 2D fracture problem. The instabilities are seen to appear at the critical crack speed of $0.48c_s$, where $c_s$ is the shear wave speed. It is worth to note that this relation is valid for perfect brittle materials only. Moreover, it was observed that, as the crack speed increases, the curvature of the area around the crack tip increases, splitting into two cracks when a critical value for crack speed is attained. In [118], a 3D study of crack branching stability is performed by means of fractographic patterns. The authors conclude that the instability is either restricted to a portion of the crack front or a quasi-2D branches.

A phase-field model is applied for damage evolution in composite materials in [29]. The evolution equation of the phase-field model was able to include difficult topological changes during damage evolution, such as void nucleation and crack branching and merging. Moreover, no meshing was required by the used phase-field model.

In [141], the formulation used in [39] is complemented by a Ginzburg-Landau type evolution equation, where an additional variable *M* is responsible for the crack propagation behaviour. If *M* is too small, the crack propagation may be delayed, while for sufficiently high values, the crack propagation is not affected by *M*. The FEM was coupled with the phase-field theory. This work was extended by [242] for dynamic brittle fracture.

Numerical aspects of the phase-field models used with finite differences, FEM and multipole expansion methods are discussed in [211].

More information about phase-field methods can be found in [38, 50, 217, 242, 253, 275].

### Configurational Force Method

Numerical implementations of brittle fracture propagation are relatively rare in the computational mechanics literature. One of the most promising numerical techniques developed within a conventional finite-element framework over the last decade is based on configurational forces. Within this setting, the most recent application of the configurational force methodology to the modelling of fracture is the work of Kaczmarczyk et al. [129], which focuses on large, hyperelastic, isotropic three-dimensional problems.

Kaczmarczyk et al.’s paper [129] is largely based on the work of Miehe and co-workers [103, 170, 171]. Miehe and Gürses [170] presented a two-dimensional large strain local variational formulation for brittle fracture with adaptive R-refinement, the simplification of this framework to small strain problems was presented by Miehe et al. [171]. The approach was extended to three-dimensions for the first time by Gürses and Miehe [103].

All of the works in this area are based on Eshelby [70, 73] and Rice’s [224] concept of material configurational forces acting on a crack tip singularity. A more general overview can be obtained from several sources [104, 105, 135, 162, 256]. Within this setting several local variational formulations have been proposed, for example see the works of [163, 258], and fracture initiation defects of the classical Griffith-type brittle fracture overcome by global variational formulations [54, 80]. Several researchers have numerically determined the material configurational forces at static fracture fronts [61, 116, 185, 255]. Before the works of Miehe and co-workers [103, 170, 171], there were several other attempts towards the implementation of fracture propagation in the configurational mechanics context, including: Mueller and Maugin [186] within the conventional finite-element context, Larsson and Fagerström [74, 143] in X-FEM and Heintz [115] within a discontinuous Galerkin (DG) setting. The framework has also recently been applied to materials with non-linear behaviour, see for example the works of Runesson et al. [229] and Tillberg and Larsson [265] on elasto-plasticity and Näser et al. [189, 190] on time-dependent materials and the review by Özenç et al. [202]. In the following a configurational force approach to modelling fracture propagation is outlined based on the notation of Kaczmarczyk et al. [129].

**Fig. 14:**
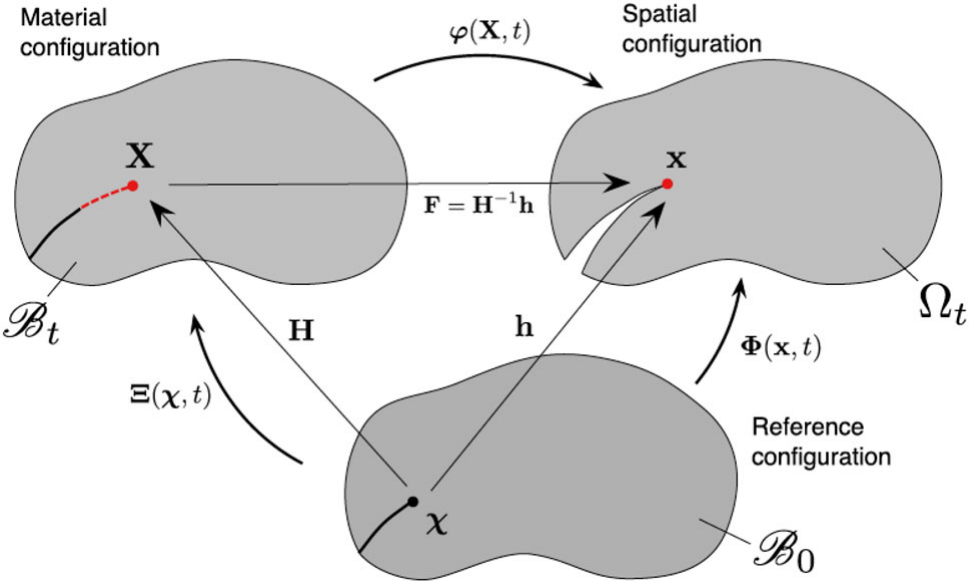
Reference, spatial and material configurations for a body with a propagating crack (from [129])

**Fig. 15:**
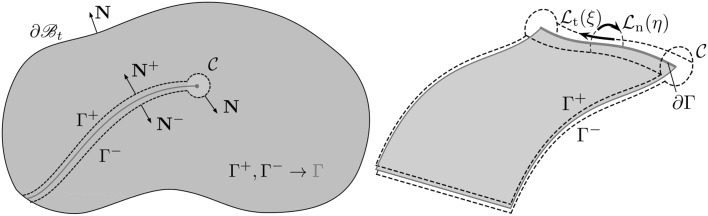
Configurational force crack (from [129])

The method can be cast within an Arbitrary Lagrangian-Eulerian (ALE) description of motion, where the deformation of the body is decoupled from the development of an advancing crack front (see Fig. 14). This approach requires the specification of three configurations: a reference state, ${\mathscr {B}}_0$; and two current states: a material configuration, ${\mathscr {B}}_{t}$, containing the evolution of the crack surface; and a spatial configuration, $\Omega _t$, containing the physical deformation of the body. A conventional finite-deformation mapping, $\varvec{\varphi }(\varvec{X},t)$, connects the spatial, $\varvec{x}$, and material, $\varvec{X}$, configurations. Similarly the material and reference, $\varvec{\Xi }$, frames are linked by a deformation mapping, $\varvec{\Xi }(\varvec{\chi },t)$, that contains the structural change of the material. The crack surface is denoted as $\Gamma \in {\mathscr {B}}_t$ and the crack front, $\partial \Gamma$, as shown in Fig.  15.

From the first law of thermodynamics, equilibrium of the crack front is governed by

77 $$\dot{\varvec{W}}\cdot (\gamma \varvec{A}_{\partial \Gamma }- \varvec{G}_{\partial \Gamma })=0,$$

where $\dot{\varvec{W}}$ is the crack front velocity, $\varvec{A}_{\partial \Gamma }$ is a kinematic state variable that defines the current crack front direction and $\gamma$ is the surface energy. The configurational force at the crack front, $\partial \Gamma$, is given by

78 $$\varvec{G}_{\partial \Gamma } = \lim _{|\mathcal {L}_n|\rightarrow 0}\int _{\mathcal {L}_n}\varvec{\Sigma }\varvec{N} {\text {d}}L,$$

where $\varvec{N}$ is the normal to the surface encircling $\partial \Gamma , \varvec{\Sigma }$ is the Eshelby stress tensor, *L* is the length $\partial \Gamma , {\mathcal {L}_n}$ is the curve orthogonal to $\partial \Gamma$ that defines the crack front encircling surface (as shown in Fig. 15). The Eshelby stress tensor, $\varvec{\Sigma }$, is defined as

79 $$\varvec{\Sigma }=\Psi (\varvec{F})\varvec{1}-\varvec{F}^{T}\varvec{P},$$

where $\Psi (\varvec{F})$ is the free-energy function, $\varvec{F}$ the deformation gradient, $\varvec{1}$ is the second order identity tensor and $\varvec{P}=\partial \Psi (\varvec{F})/\partial \varvec{F}$ is the first Piola-Kirchhoff stress.

As noted by Kaczmarczyk et al. [129], three possible solutions to Eq. (77): zero crack growth with $\dot{\varvec{W}}=0$; force balance $(\gamma \varvec{A}_{\partial \Gamma }- \varvec{G}_{\partial \Gamma })=0$; or that the crack front velocity is orthogonal to $(\gamma \varvec{A}_{\partial \Gamma }- \varvec{G}_{\partial \Gamma })$. However, there is insufficient information in Eq. (77) to dictate the evolution of the crack front. Such an evolution law can be obtained by considering the second law of thermodynamics, supplemented by a material constitutive law and the principal of maximum energy dissipation.

Starting from a Griffith-type criterion for crack growth

80 $$\varvec{G}_{\partial \Gamma }\cdot \varvec{A}_{\partial \Gamma }-g_c/2 \le 0,$$

where $g_c$ is a material parameter controlling the critical threshold of energy release per unit area. Combining this with the principal of maximum dissipation, and through the application of Lagrange multipliers, it is possible to arrive at the condition that

81 $$\gamma \varvec{A}_{\partial \Gamma }=\varvec{G}_{\partial \Gamma } \qquad {\text {and}} \qquad 2\gamma =g_c.$$

Therefore, the direction of crack propagation is constrained to be coincident with the configurational force direction. In addition, the configurational force approaches based on the work of Miehe and co-workers [103, 129, 170, 171] utilise R-adaptive mesh alignment. This method aligns the propagating crack front with the direction of the configurational force by modifying the position of the node(s) attached to the element faces to be split.

In the work of Kaczmarczyk et al. [129], this fracture methodology was combined with a mesh quality control algorithm based on the work of Scherer et al. [240]. Within this, the nodal positions of the elements are modified based on a shape-based (volume to length) measure of element quality through the determination of a pseudo force vector. This pseudo force features in the discretised *material* nodal force equilibrium equation and is solved using a Newton-Raphson process. Note, that this modification to the discrete equilibrium equation only influences the stability of the solution and not the crack propagation criterion [129]. This mesh quality control procedure reduces the progressive degradation of the solution with fracture propagation.

Kaczmarczyk et al. [129] note that their approach could easily be extended to anisotropic materials. However, one limitation of the approach is that it is currently unable to capture non-smooth crack kinking [171]. Also, crack branching and multiple crack coalescence has yet to have been demonstrated, or even formulated.

### Discrete Element Method (DEM)

**Fig. 16:**
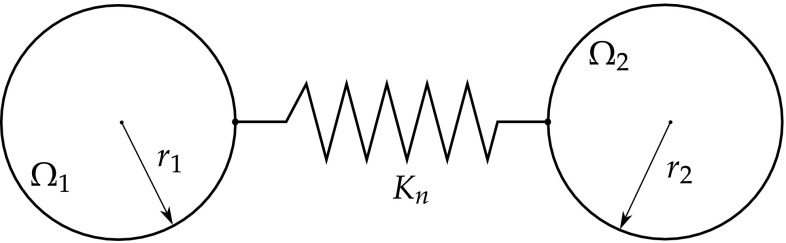
Bonded-particle approach

**Fig. 17:**
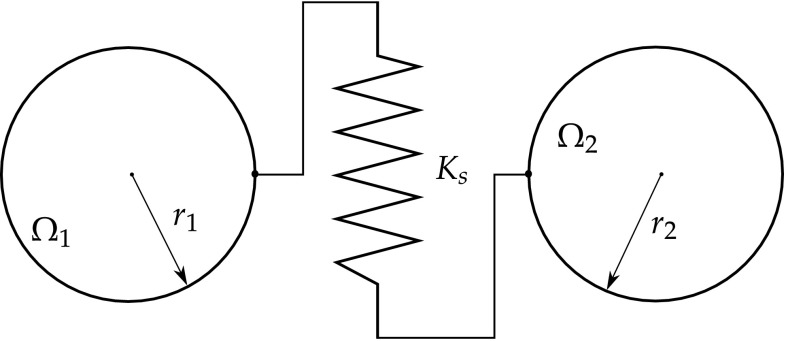
Parallel-contact approach

The discrete element method (DEM) has been initially developed for materials which have particle-like behaviour, such as soil and rocks [146]. The method was formally proposed by Cundall and Hart [53] and consisted of modelling of the interaction between elements using contact. This was later called the bonded-particle approach and is illustrated in Fig. 16 for two arbitrary bodies $\Omega _1$ and $\Omega _2$ having a normal contact stiffness $K_n$. However, one of the main restrictions of this bonded-particle approach is that it did not allow rotations, and therefore does not consider momentum. To overcome this restriction, shear contact stiffness $K_s$ has been introduced to the formulation and can be seen in Fig. 17.

The DEM is characterised by the following properties [30, 146, 215]:

- Finite displacements and rotations of the bodies is permitted, which includes complete detachment;
- New contacts (or the absence thereof) are recognised automatically as the calculation progresses.

In practice, DEM is used in problems with a large number of elements, each element representing a body in contact. The formulation itself can be quite simplified compared to other discretisation methods, but it allows the simulation of complex behaviour, including material heterogeneities.

The DEM can be decomposed into several subclasses, which differ in some aspects such as the contact treatment, material models, number of interacting bodies, fracturing, and integration schemes [30].

In this framework, each element is a particular body which can be in contact with a number of surrounding elements. This implies that contact detection is one of the main problems that can arise, since missing a contact between elements can result in non representative behaviour of the model. Moreover, inspecting the elements for possible contact can require large amounts of computational processing time. The most common contact search algorithms are based on so-called body based search, where the vicinity of a given discrete element is searched for possible contact, and repeated after a number of iterations to check if the elements are still in contact. The Region Search algorithm [263] is an example of this kind of contact detection. Other contact detection algorithms use space search rather than a body search, and some examples are based on binary trees [30, 36, 208].

The next step is to obtain the contact forces. The calculation is usually performed with penalty based methods or Lagrange multiplier based methods. A review of contact algorithms evaluation can be found in [112].

The modelling of fracture using DEM has been mostly confined to element interfaces, where the breakage of the link between elements determines the appearance or propagation of the damage [30]. Particles can be bonded into clusters, where the bond stiffnesses are the equivalent to the continuum strain energy. Bond failure is assumed when the strength has exceed the maximum tension the bond can handle. Consistent breakage of the particle bonds define the fracture shape in the material. In [18, 187], a combination of the FEM with DEM has been used to model fracture starting from a continuum representation of the finite elements, and as the damage appears it is restated in the discrete element framework. A multifracture FEM/DEM scheme has been proposed by [212], where sliver elements arising from poor intra-element fracturing were avoided using local adaptive mesh refinement.

Discontinuous deformation analysis (DDA) is a variation of the DEM proposed originally by Shi and Goodman [246] to simulate the dynamics, kinematics, and elastic deformability of a system contacting rock blocks. While each block is treated separately in DEM, in DDA the total energy of the system is minimised in order to obtain a solution; a linear system of equations is obtained, resembling the finite element formulation. In fact, displacements and strains are taken as variables and the stiffness matrix of the model is assembled by differentiating several energy contributions including block strain energies, contacts between blocks, displacement constraints and external loads [146]. In the basic DDA implementation, each block is simply deformable as the strain and stress fields are constant over the entire block area, while the contacts are solved using regular contact algorithms that allow interpenetration between bodies [112]. To conclude, DDA is an implicit formulation while DEM uses an explicit procedure to solve the equilibrium equations. DDA has been used extensively in rock mechanics applications, as can be seen in [113, 114, 155, 267] for example.

The influence of the bond parameters defined at the microscale and how they affect the response on the macroscale are analysed in detail in [49] for rock model analysis. It is shown that using a clumped-particle model, i.e. the particles rotate in a cluster instead of each particle being allowed to rotate, can reduce the limitations of the model, such as the overestimated ratio between tensile and compressive strengths, and the friction angles of the failure envelope.

A combined Lattice Boltzmann method (LBM) and DEM have been used to simulate fluid-particle interactions by [76]. The fluid field is solved by an extended 3D LBM with a turbulence model, while particle interactions are modelled using the DEM. Simulation results have matched experimental measurements.

There are available codes for the DEM, as the universal distinct element code (UDEC) [124], the ELFEN [218], the Yade [138] and Y-Geo [159]. More information on the discrete element framework can be found on [30, 146] and some applications in [33, 127, 128].

## Peridynamics

We will now introduce a new numerical method called peridynamics, which appears to be very promising for fracking problems. The main difference between the peridynamic theory and classical continuum mechanics is that the former is formulated using integral equations as opposed to derivatives of the displacement components. This feature allows damage initiation and propagation at multiple sites, with arbitrary paths inside the material, without resorting to special crack growth criteria. In the peridynamic theory, internal forces are expressed through non-local interactions between pairs of material points within a continuous body, and damage is a part of the constitutive model. Interfaces between dissimilar materials have their own properties, and damage can propagate when and where it is energetically favourable for it to do so.

### Definitions

The peridynamics formulation was first developed by Silling [248], where he tried to overcome the limitation of current theories dealing with discontinuity, such as in fracture mechanics problems. The main argument was that the difficulty of existing theories was due to the presence of partial derivatives in the formulation to represent the displacement and forces, making necessary specific approaches to eliminate the singularities which would arise. Silling proposed a new formulation based on particular interactions as in molecular dynamics, but applied to continuum mechanics. The term *peridynamics* was adopted to describe this formulation, and it comes from the Greek roots for near and force. The pairwise interaction between two particles can be defined as [249]

82 $$\rho \ddot{{\mathbf {u}}}({\mathbf {x}},t) = \int _{{\mathscr {H}}} {\mathbf {f}}({\mathbf {u}}({\mathbf {x}}',t)-{\mathbf {u}}({\mathbf {x}},t),{\mathbf {x}}' - {\mathbf {x}})\ dV_{x'} + {\mathbf {b}}({\mathbf {x}},t)$$

where $\rho$ is the mass density, ${\mathbf {f}}$ is the pairwise force function that the particle ${\mathbf {x}}'$ exerts on the particle ${\mathbf {x}}, {\mathscr {H}}$ is the neighbourhood of ${\mathbf {x}}$, ${\mathbf {u}}$ is the displacement vector field, ${\mathbf {b}}$ is a prescribed force vector field (per unit volume). It is usual to adopt the relative position $\varvec{\xi }$ of the two particles in the reference configuration as

83 $$\varvec{\xi }= {\mathbf {x}}' - {\mathbf {x}}$$

Analogously, the relative displacement $\varvec{\eta }$ is stated as

84 $$\varvec{\eta }= {\mathbf {u}}({\mathbf {x}}',t)- {\mathbf {u}}({\mathbf {x}},t)$$

The current relative position can be easily given as $\varvec{\eta }+ \varvec{\xi }$. The function ${\mathbf {f}}$ must satisfy two conditions

85 $${\mathbf {f}}(-\varvec{\eta },-\varvec{\xi }) = -f(\varvec{\eta },\varvec{\xi })$$

which represents Newton’s third law and enforces conservation of linear momentum, and

86 $$(\varvec{\xi }+ \varvec{\eta }) \times {\mathbf {f}}(\varvec{\eta },\varvec{\xi }) = {\mathbf {0}},\quad \forall \varvec{\eta }, \varvec{\xi }$$

which assures conservation of angular momentum.

The interaction between particles is defined as a bond, which in continuum mechanics could also be considered as a spring connecting two particles. This definition is fundamentally the difference between the classical theory and peridynamics, where the main idea is the direct contact between two particles. The area of influence of a particle is defined as the horizon $\delta$ and is stated as

87 $$\forall |\varvec{\xi }| > \delta \Rightarrow {\mathbf {f}}(\varvec{\eta }, \varvec{\xi }) = {\mathbf {0}}.$$

**Fig. 18:**
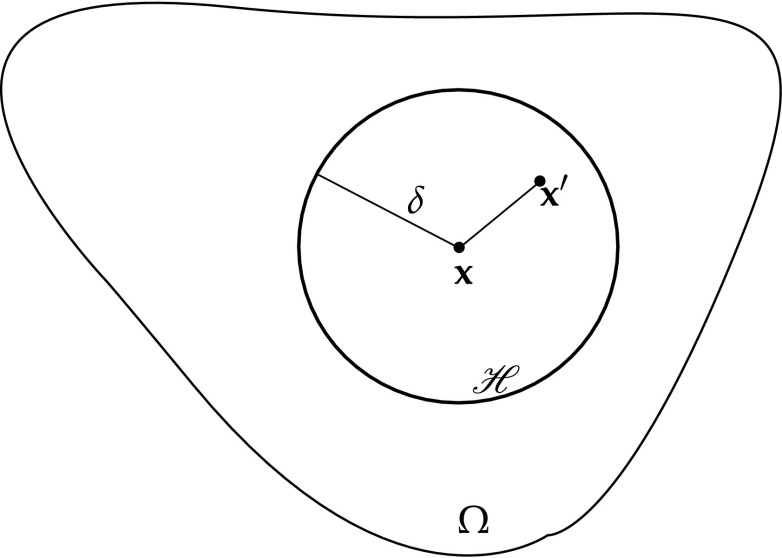
Particle interaction in a peridynamics solid

Figure 18 illustrates the horizon $\delta$ in an arbitrary body. Outside the horizon $\delta$, a particle has no influence on the other particles. For this reason, the peridynamics formulation is considered as a non-local model.

A material is microelastic if the pairwise function can be obtained through derivation of a scalar micropotential *w* such as

88 $${\mathbf {f}}(\varvec{\eta },\varvec{\xi }) = \frac{\partial w}{\partial \varvec{\eta }}(\varvec{\eta }, \varvec{\xi }) \quad \forall \varvec{\eta }, \varvec{\xi }$$

The micropotential *w* is the energy present in a single bond (in terms of energy per unit volume squared). Thus, the local strain energy density is defined as

89 $$W = \frac{1}{2} \int _{{\mathscr {H}}} w(\varvec{\eta }, \varvec{\xi }) dV_{\xi }$$

where the factor 1/2 is present since each particle possesses half of the energy of the bond between them. If a material is microelastic, then every pair of particles ${\mathbf {x}}$ and ${\mathbf {x}}'$ is connected by a spring. The force in the spring depends only on the distance between the particles in the deformed configuration. Hence, there is a scalar function $\hat{w}$ such that

90 $$\hat{w}(y,\varvec{\xi }) = w(\varvec{\eta }, \varvec{\xi })\quad \forall \varvec{\eta }, \varvec{\xi }, \quad y = |\varvec{\eta }+ \varvec{\xi }|$$

From Eqs. (88) and (90), the pairwise function ${\mathbf {f}}$ is restated as

91 $${\mathbf {f}}(\varvec{\eta },\varvec{\xi }) = \frac{\varvec{\xi }+ \varvec{\eta }}{|\varvec{\xi }+ \varvec{\eta }|} f(|\varvec{\xi }+ \varvec{\eta }|,\varvec{\xi })\quad \forall \varvec{\eta }, \varvec{\xi }$$

with

92 $$f(y, \varvec{\xi }) = \frac{\partial \hat{w}}{\partial y}(y, \varvec{\xi })\quad \forall y, \varvec{\eta }$$

From Eqs. (82) and (91), the peridynamics model is fully defined for a non-linear microelastic material. However, a linearised theory of the peridynamics microelasticity can be defined as

93 $${\mathbf {f}}(\varvec{\eta },\varvec{\xi }) = {\mathbf {C}}(\varvec{\xi })\varvec{\eta }\quad \forall \varvec{\eta }, \varvec{\xi }$$

where ${\mathbf {C}}$ is the material’s micromodulus function. It will be seen that the micromodulus has similar function to the material constitutive law. For more details, see reference [248].

Boundary conditions in peridynamics are not completely alike to the classical theory. Although the essential boundary condition is still present (displacements), there are no natural boundary conditions (tractions) in the peridynamics framework. Forces at the surface of a body must be applied as body forces ${\mathbf {b}}$ acting through the thickness of some layer under the surface. Usually, the thickness is taken to be the horizon $\delta$. The displacement boundary conditions also have to be imposed as a volume rather than a surface. For more details see [248].

### Constitutive Modelling

We assume that the bond force *f* depends only on the bond stretch *s*, defined as

94 $$s = \frac{|\varvec{\xi }+ \varvec{\eta }|-|\varvec{\xi }|}{|\varvec{\xi }|} = \frac{y-|\varvec{\xi }|}{|\varvec{\xi }|}$$

As expected, *s* is positive only when the bond is under tension. Failure is introduced into the peridynamics model through breakage of the bonds connecting two particles over some stretching limit. Once a bond fails, it never becomes reconnected (i.e. no healing is considered). An example of a history dependent model is given by the prototype microelastic brittle (PMB) material, and is given by

95 $$f(y(t),\varvec{\xi }) = g(s(t,\varvec{\xi }))\mu (t,\varvec{\xi })$$

where $g(s)=c s, c$ is a constant and $\mu$ is a history-dependent scalar-valued function, assuming either the values 0 or 1 according to

96 $$\mu (t,\varvec{\xi }) = \left\{ \begin{array}{ll} 1 & \hbox {if }s(t',\varvec{\xi }) < s_0\hbox { for all }0 \le t' \le t, \\ 0 & \hbox {otherwise} \end{array} \right.$$

In this case, $s_0$ is the critical stretch for bond failure. The local damage at a point can be defined as

97 $$\varphi ({\mathbf {x}},t) = 1 - \frac{\int _{{\mathscr {H}}}\mu ({\mathbf {x}},t,\varvec{\xi })\ dV_\xi }{\int _{{\mathscr {H}}} dV_\xi }$$

where ${\mathbf {x}}$ has been included as a reminder that the history model also depends on the position in the body. One can see that $0\le \varphi \le 1$, 0 representing the undamaged state and 1 representing full break of all the bonds of a given particle to all other particles inside the horizon $\delta$. The broken bonds will eventually lead to some softening material response, since failed bonds cannot sustain any load.

There are only two parameters that define the PMB material, the spring constant *c* and the critical stretch $s_0$. Assuming $\varvec{\eta }= s\varvec{\xi }$ and substituting in Eq. (89), the local strain energy can be expressed as

98 $$W = \frac{\pi c s^2\delta ^4}{4}$$

This relation must be identical to its equivalent in the classical theory, $W = 9ks^2/2$, where *k* represents the material bulk modulus [249]. The spring constant of the PMB material model is obtained as

99 $$c = \frac{18 k}{\pi \delta ^4}$$

Now we describe the bond breakage formulation. Let the work $G_0$ necessary to break all the bonds per unit fracture area be given as

100 $$G_0 = \int _0^\delta \int _0^{2\pi } \int _z^\delta \int _0^{cos^{-1}z/\xi } (c s_0^2\xi /2)\xi ^2 \sin {\varphi }\ d\varphi d\xi d\theta dz$$

**Fig. 19:**
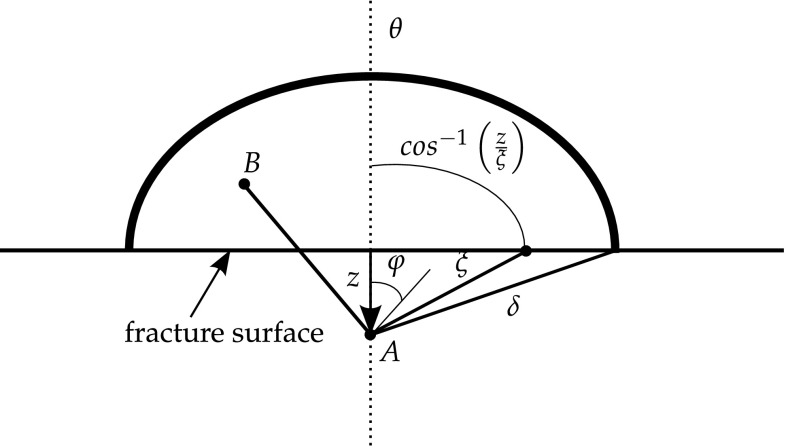
Fracture energy evaluation

The elements of Eq. (100) are depicted in Fig. 19. Equation (100) is the energy to break all points *A*, where $0\le z \le \delta$ from the points *B*. After evaluation of the integrals we obtain

101 $$G_0 = \frac{\pi c s_0^2 \delta ^5}{10}$$

### Anisotropic Materials in Peridynamics

The peridynamics formulation was initially presented for isotropic materials, in order to make some simplifying assumptions. It is expected then that the spring stiffness of the bonds does not vary over the direction of $\varvec{\xi }$. It was demonstrated in detail in [248] that for isotropic materials, the Poisson’s ratio in the peridynamics formulations is constrained to take the constant value of 1/4. The constant Poisson’s ratio is a consequence of the Cauchy relation for a solid composed of a lattice of points that interact only through a central force potential [153].

Refinements of the peridynamics theory can allow the dependence of strain energy density on local volume change in addition to two-particle interactions [154].

A composite material is formed by different materials, commonly a brittle and stiff material (fibre) embedded into a ductile one (matrix). In [201], the micromodulus ${\mathbf {C}}$ is redefined in order to accommodate the new variables arising from the material’s anisotropy, including the fibre and matrix bonds for a laminate, and the shear and interlayer bonds present between two different laminates. However, in real composite materials, the fibre and matrix present properties vary significantly with the direction, which was not the case in this work. Instead, different isotropic materials were employed to form the composite fibre and matrix. In [122], the fracture in fibre-reinforced composites is tackled with more attention to the material modelling, where the differences between the fibre and matrix bonds are specifically defined. Moreover, the effect of arbitrary fibre orientation in the peridynamic model is taken into account, and it is shown that for a given particle ${\mathbf {x}}$, the number of fibre bond particles within the horizon $\delta$ can vary considerably, which leads to large variation of the strain energy density, the parameter which describes the bond stiffness. To consider this modelling issue, a semi-analytical model was deduced for fibre orientation of $45^\circ$, and also for random fibre orientation.

**Fig. 20:**
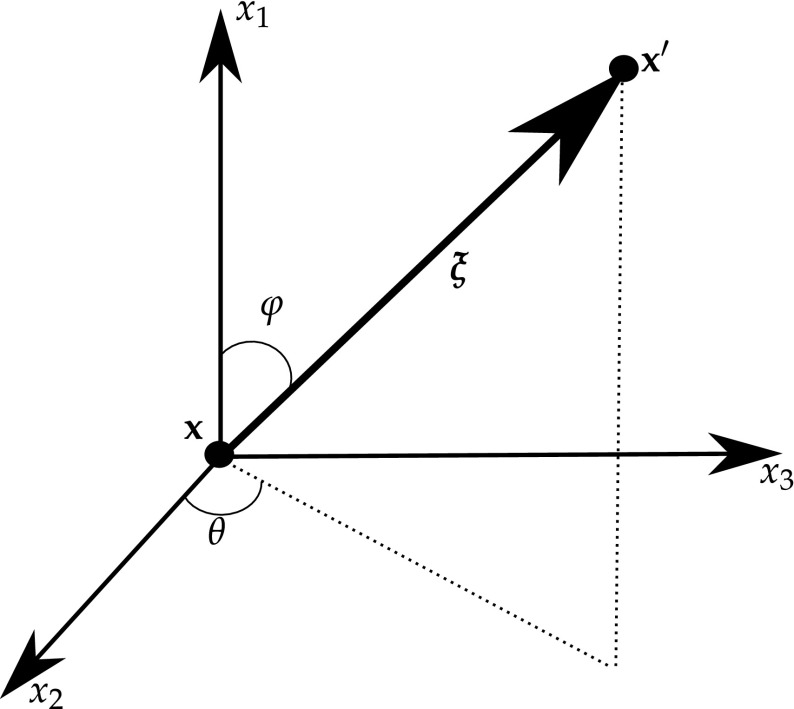
Direction of a peridynamic bond in the principal axes

A recent work [95] has deduced a peridynamic formulation for orthotropic media. The micromodulus ${\mathbf {C}}$ is defined in terms of the orientation of the angles $\varphi$, as illustrated in Fig. 20. The dependency on the $\theta$ orientation can be suppressed since the material properties do not change over $\theta$ for a transversely isotropic material. After some mathematical manipulation, the new definition of the micromodulus is given as

102 $$c(\varphi ) = \sum _{n=0}^\infty A_{n0}P_n^0 (\cos \varphi )$$

where $A_{n0}$ represents constant coefficients and $P_n^m$ are the associated Legendre functions of degree *n* and order *m*

103 $$P_n^m(\cos \varphi ) = \frac{(-1)^m}{2^n n!}(1-\cos {^2\varphi })^{m/2}\frac{d^{n+m}}{d(\cos \varphi )^{n+m}}(\cos {^2 \varphi }-1)^n$$

Equation (103) can be further simplified into

104 $$c(\varphi ) = A_{00}+A_{20}P_2^0(\cos \varphi ) = A_{00} + A_{20}\frac{1}{2}(3\cos {^2\varphi }-1)$$

Assuming $c(0)=c_1$ and $c(\pi /2)=c_2$, it can be shown that Eq. (104) is also equivalent to

105 $$c(\varphi ) = c_2 + (c_1-c_2)\cos {^2\varphi }$$

where $c_1$ and $c_2$ are constants of the material model and are given by

106 $$c_1= \frac{15.41C_{11}-7.41C_{22}}{\pi \delta ^3 t}$$

107 $$c_2= \frac{8.08C_{22}-0.08C_{11}}{\pi \delta ^3 t}$$

108 $$C_{12}= C_{66} = 0.059C_{11}+0.274C_{22}$$

where $C_{11}, C_{22}, C_{16}$ and $C_{66}$ are elements of the constitutive matrix given in the Voigt notation. Note that an orthotropic material has only 2 independent material constants in the peridynamic model instead of the normal 4 independent constants. This restriction is used linked to the fact that a point is only able to interact to another one individually, while in the classical theory this condition does not apply (a disturbance in a continuous point will automatically induce some disturbance on the points around the body). This restriction on peridynamics theory has been addressed by Silling et al. [250] and will be detailed in the next section.

The critical bond stretch also depends on the direction of $\varvec{\xi }$ and is given by

109 $$s_0^2(\varphi ) = B_{00}+B_{20}P_2^0(\cos \varphi )+B_{40}P_4^0(\cos \varphi )+B_{60}P_6^0(\cos \varphi )+B_{80}P_8^0(\cos \varphi )$$

where $B_{n0}$ are constants and are detailed in [95]. The critical strain energy release rates for mode I crack propagation in the planes normal to the principal axes 1 ($G_{Ic1}$) and 2 ($G_{Ic2}$) can be obtained from the following relations

110 $$G_{Ic1}= \int _0^\delta \int _z^\delta \int _{-\cos {^{-1}(z/\xi )}}^{\cos {^{-1}(z/\xi )}} \left[ \frac{c(\varphi )s_0^2(\varphi )\xi }{2} t\xi \ d\varphi d\xi dz \right]$$

111 $$G_{Ic2}= \int _0^\delta \int _z^\delta \int _{-\sin {^{-1}(z/\xi )}}^{\pi -\sin {^{-1}(z/\xi )}} \left[ \frac{c(\varphi )s_0^2(\varphi )\xi }{2} t\xi \ d\varphi d\xi dz \right]$$

After integration of Eqs. (110) and (111), the critical stretches $s_{01}$ and $s_{02}$ are given by

112 $$s_{01}^2= \frac{500[(4G_{Ic1}-11G_{Ic2})c_1+(112G_{Ic1}-72G_{Ic2})c_2]}{t\delta ^4(71c_1^2+3168c_1 c_2+994c_2^2)}$$

113 $$s_{02}^2= \frac{500[(31.5G_{Ic1}-5G_{Ic2})c_1+(11G_{Ic1}-4G_{Ic2})c_2]}{t\delta ^4(71c_1^2+3168c_1 c_2+994c_2^2)}$$

The fracture behaviour of the material is fully defined by using the mode I energy release rates. Hence, mode II energies are not independent from mode I, which is another consequence of the bond-based peridynamic theory.

An important issue has been highlighted in [95, 201], concerning the use of “unbreakable” bonds near to the regions where a traction boundary condition is applied. The possible reason for this would be crack initiation and propagation close to these regions, due to the high stresses that could be present. It is important to understand the physics of the analysed problem properly in order to use this type of assumption during a peridynamic simulation.

### State-based Formulation

The peridynamics formulation assumes that any pair of particles interacts only through a central potential which is independent of all the other particles surrounding it. This oversimplification has led to some restrictions of the material’s properties, such as the aforementioned fixed Poisson’s ratio of 1/4 for isotropic materials. Also, the pairwise force is responsible for modelling the constitutive behaviour of the material, which is originally dependent on the stress tensor. To overcome this limitation, Silling et al. [250] have extended the peridynamics formulation to include vector states. The vector states allow us to consider not only a particle, but a group of particles in the peridynamics framework. Moreover, the direction of the vector states would not be conditioned to be in the same direction of the bond, as in the bond-based theory. This property is fundamental to consider truly anisotropic materials.

Let $\underline{{\mathbf {A}}}$ be a vector state. Then, for any $\varvec{\xi }\in {\mathscr {H}}$, the value of ${\underline{\mathbf {A}}} \langle \varvec{\xi }\rangle$ is a vector in $\mathbb R^3$, where brackets indicate the vector on which a state operates. The set of all vector states is denoted ${\mathscr {V}}$. The dot product of two vector states $\underline{{\mathbf {A}}}$ and $\underline{\mathbf {B}}$ is defined by

114 $$\underline{\mathbf {A}} \cdot \underline{\mathbf {B}} = \int _{{\mathscr {H}}} \underline{\mathbf {A}}_i \langle \varvec{\xi }\rangle \underline{\mathbf {B}}_i \langle \varvec{\xi }\rangle \ dV_{\xi }$$

The concept of a vector state is similar to a second order tensor in the classical theory, since both map vectors into vectors. Vector states may be neither linear nor continuous functions of $\varvec{\xi }$. The characteristics of the vector states are listed in [250], and they imply the vector states mapping of ${\mathscr {H}}$ may not be smooth as in the usual peridynamic model, including the possibility of having a discontinuous surface.

In the state theory, the equation of motion (82) is redefined as

115 $$\rho ({\mathbf {x}}) \ddot{{\mathbf {u}}}({\mathbf {x}},t) = \int _{{\mathscr {H}}} \{ \underline{{\mathbf {T}}}[{\mathbf {x}},t] \langle {\mathbf {x}}' - {\mathbf {x}} \rangle \underline{{\mathbf {T}}}[{\mathbf {x}}',t] \langle {\mathbf {x}} - {\mathbf {x}}' \rangle \}\ dV_{{\mathbf {x}}'} + {\mathbf {b}}({\mathbf {x}},t)$$

with $\underline{{\mathbf {T}}}$ as the force vector state field, and square brackets denote that the variables are taken in the state vector framework.

To ensure balance of linear momentum, $\underline{{\mathbf {T}}}$ must satisfy the following relation for any bounded body ${\mathscr {B}}$

116 $$\int _{{\mathscr {B}}} \rho \ddot{{\mathbf {u}}}({\mathbf {x}},t)\ dV_{{\mathbf {x}}} = \int _{{\mathscr {B}}} {\mathbf {b}}({\mathbf {x}},t)\ dV_{{\mathbf {x}}}$$

The balance of angular momentum for a bounded body ${\mathscr {B}}$ is also required

117 $$\int _{{\mathscr {B}}} {\mathbf {y}}({\mathbf {x}},t) \times (\rho \ddot{{\mathbf {u}}}({\mathbf {x}},t) - {\mathbf {b}}({\mathbf {x}},t))\ dV_{{\mathbf {x}}} = 0, \quad \forall t \ge 0,\ {\mathbf {x}} \in {\mathscr {B}}$$

where

118 $${\mathbf {y}}({\mathbf {x}},t) = {\mathbf {x}} + {\mathbf {u}}({\mathbf {x}},t)$$

The deformation vector state field is stated as

119 $$\underline{{\mathbf {Y}}}[{\mathbf {x}},t]\langle \varvec{\xi }\rangle = {\mathbf {y}}({\mathbf {x}} + \varvec{\xi },t)-{\mathbf {y}}({\mathbf {x}},t), \quad \forall {\mathbf {x}} \in {\mathscr {B}},\ \varvec{\xi }\in {\mathscr {H}},\ t \ge 0$$

The non-local deformation gradient for each individual node is given by

120 $${\mathbf {B}}({\mathbf {x}})= \left[ \int _{{\mathscr {H}}} \omega (|\xi |)(\xi \otimes \xi )\ dV_{\xi } \right] ^{-1}$$

121 $${\mathbf {F}}({\mathbf {x}})= \left[ \int _{{\mathscr {H}}} \omega (|\xi |)(\underline{{\mathbf {Y}}}(\xi ) \otimes \xi )\ dV_{\xi } \right] . {\mathbf {B}}({\mathbf {x}})$$

where $\otimes$ denotes the dyadic product of two vectors, and $\omega (|\xi |)$ is a dimensionless weight function, used to increase the influence of the nodes closes to ${\mathbf {x}}$. The use of this factor is still under study [280], but the assumption of $\omega (|\xi |)=1$ has been seen to provide good results.

The discretisation of Eqs. (120) and (121) can be expressed as a Riemann sum as [280]

122 $${\mathbf {B}}({\mathbf {x}}_j)= \left[ \sum _{n=1}^m \omega (|{\mathbf {x}}_n- {\mathbf {x}}_j|)( ({\mathbf {x}}_n- {\mathbf {x}}_j) \otimes ({\mathbf {x}}_n- {\mathbf {x}}_j))V_n \right] ^{-1}$$

123 $${\mathbf {F}}({\mathbf {x}}_j)= \left[ \sum _{n=1}^m \omega (|{\mathbf {x}}_n- {\mathbf {x}}_j|)(\underline{{\mathbf {Y}}}\langle {\mathbf {x}}_n- {\mathbf {x}}_j \rangle \otimes ({\mathbf {x}}_n- {\mathbf {x}}_j))V_n \right]$$

where *m* is the number of nodes with the horizon of node *j*. ${\mathbf {x}}_j$ must be connected to at least three other nodes in the system to ensure that ${\mathbf {B}}({\mathbf {x}}_j)$ will not be singular.

In state vector peridynamics, there are two ways to determine how the force state depends on the deformation near a given point. The first consists of formulating a constitutive model in terms of the force vector $\underline{{\mathbf {T}}}$ and the deformation state $\underline{{\mathbf {Y}}}[{\mathbf {x}},t]$. In this case, the force state is defined as

124 $$T = \nabla W$$

where *W* is the strain energy density and $\nabla$ indicates the Fréchet derivative, which is defined as any infinitesimal change in the deformation state $d\underline{{\mathbf {Y}}}$ resulting in a change of the strain energy density *dW* such as

125 $$dW = W(\underline{{\mathbf {Y}}}+d\underline{{\mathbf {Y}}}) - W(\underline{{\mathbf {Y}}}) = \int _{H_x} \underline{{\mathbf {T}}} \langle \xi \rangle . d\underline{{\mathbf {Y}}} \langle \xi \rangle\ dV_\xi$$

with $H_x$ being a sphere centred at the point ${\mathbf {x}}$ with radius equal to the horizon $\delta$. Note that the Fréchet derivative can be seen as an equivalent of the tensor gradient in classical theory.

The second approach to relating the force and deformation in a state vector framework is to adopt a stress-strain model as an intermediate step [42, 280]. For a strain energy density $W({\mathbf {F}})$, the stress tensor can be expressed as

126 $$[\sigma ]^t = \frac{\partial W}{\partial {\mathbf {F}}}$$

The force vector is redefined as [250]

127 $$T = \nabla W = \frac{\partial W}{\partial {\mathbf {F}}}\nabla {\mathbf {F}}$$

After evaluation of the Fréchet derivative, the force vector can be defined explicitly as

128 $$\underline{{\mathbf {T}}}\langle {\mathbf {x}}' - {\mathbf {x}} \rangle = \omega (|{\mathbf {x}}' - {\mathbf {x}}|)[\sigma ({\mathbf {F}})]^t. {\mathbf {B}}. ({\mathbf {x}}' - {\mathbf {x}})$$

The processing of mapping a stress tensor as a peridynamic force state is the inverse of the process of approximating the deformation state by a deformation gradient tensor. A peridynamic constitutive model that uses stress as an intermediate quantity results in general in bond forces which are not parallel to the deformed bonds. This type of modelling was called “non-ordinary” by Silling [250].

### Numerical Discretisation

The discretisation of the peridynamics model is quite straightforward. Equation (82) can be rewritten as a finite sum

129 $$\rho {\ddot{\mathbf {u}}}_i^n = \sum _p {\mathbf {f}}({\mathbf {u}}_p^n - {\mathbf {u}}_i^n,{\mathbf {x}}_p-{\mathbf {x}}_i) V_p + {\mathbf {b}}_i^n$$

where *n* is the time step and subscripts denote the node number, i.e., ${\mathbf {u}}_i^n = {\mathbf {u}}({\mathbf {x}}_i,t^n), V_p$ is the volume of node *p*. Equation (129) is taken over all *p* nodes which satisfy $|{\mathbf {x}}_p-{\mathbf {u}}_i|\le \delta$. The grid spacing $\Delta x$ is also an important parameter in the peridynamics discretisation.

The discretised form of the linearised peridynamics model is given by

130 $$\rho {\ddot{\mathbf {u}}}_i^n = \sum _p {\mathbf {C}}({\mathbf {u}}_p^n - {\mathbf {u}}_i^n,{\mathbf {x}}_p-{\mathbf {x}}_i) V_p + {\mathbf {b}}_i^n$$

The displacements ${\mathbf {u}}_i^n$ are obtained using an explicit central difference formulation,

131 $$\ddot{{\mathbf {u}}}_i^n = \frac{{\mathbf {u}}_{i}^{n+1}-2{\mathbf {u}}_{i+1}^n+{\mathbf {u}}_i^{n-1}}{\Delta t^2}$$

with $\Delta t$ as the time step. Some studies of the stability of the numerical discretisation were described in [154, 249]. It has been established that the time step must not exceed a certain value in order for the numerical discretisation to be stable. Moreover, the error associated with the discretisation depends on the time step with ($\mathcal {O}(\Delta t)$) and the grid spacing with ($\mathcal {O}(\Delta x^2)$),

132 $$\Delta t < \sqrt{\frac{2\rho }{\sum _p V_p |{\mathbf {C}}(x_p-x_i)| }}$$

Convergence in peridynamics is affected by two parameters: the grid spacing $\Delta x$ and the horizon $\delta$. Reducing the horizon size for a fixed grid spacing will lead to the peridynamics solution approximating the solution using classical theory. However, fixing the horizon size while increasing the grid spacing will lead to the exact non-local solution for that particular horizon size [122]. As for domain discretisation methods, it is important to balance the size of the horizon so the damage features in the analysed body are properly considered, and the grid spacing should be sufficiently small for the results to converge to the non-local solution. Usually, it ranges from 1/3 to 1/5 of the size of the horizon.

In recent works, the peridynamics formulation is used conjointly with other discretisation methods, such as meshless formulation [249] and finite element formulation [154].

In [201], peridynamics is used only to obtain the prediction of failure of the composite material, where an FEM code is employed to solve the global problem. This type of combined approach is often necessary since the peridynamic formulation can demand significant computational power, a common problem in molecular dynamics simulations as well.

## Conclusions and Prospective Work

We have seen that the hydraulic fracture problem presents several characteristics which makes its study complicated: the shale is not a homogeneous material, it is not isotropic, the nanoporosity may retard crack propagation as the fluid penetrates the rock, and a large fracture network has to be considered in which cracks develop at multiple length scales, all of which can greatly increase the computational solving time. Moreover, most current analytical and numerical methods do not take into account crack branching, a key factor in order to obtain a correct estimation of the extended fracture network.

The current fracture models for brittle rocks and fracking have been useful as a first step in offering a more realistic fracking model. There are of course other limitations attached to each of the numerical models discussed earlier: for instance, in cohesive models, the cohesive zone model is not a parameter to be found, so the crack propagation path is already known a priori. Most works on X-FEM and BEM models consider that the crack propagation path is unique; only recently have some works appeared considering crack branching [184, 244, 285].

Fracking models developed so far have not considered the full complexity of shale rocks. Ulm and co-workers [268–270] have established that shales are likely to be transversely isotropic materials, with the direction perpendicular to the bedding planes taken as the symmetry axis. This is mainly due to the deposition process. It was also stated that the shale anisotropy is due more to the interaction between the particles than the elastic behaviour of the shale components.

It was seen in [139] that the fluid penetrating the crack may retard crack propagation, so the material’s porosity has to be taken into account in the numerical model.

### Future Works

The main challenges researchers are facing with respect to the development of a new numerical formulation for modelling hydraulic fracture are: (1) the multiscale characteristic of the fracking in shale rocks, and (2) the requirement for the numerical method to deal with a large number of cracks simultaneously propagating and possibly branching.

For crack propagation and crack branching, the peridynamics formulation has been shown to have excellent results. A few issues have been raised about the method, such as how to choose the grid spacing (interval between particles) and the horizon (area of influence of a given particle). Even though an orthotropic formulation for 2D materials was developed by [95], there are some limitations over these formulations, since a direct bond force formulation is used. To overcome this limitation, a state-based formulation for anisotropic materials should be developed.

A multiscale model must be able to consider how a crack entering the RVE interacts with the voids that are present. Moreover, there must be a coupling between the microscale (anisotropic) and the macroscale (transversely isotropic). The peridynamics formulation could be used to model the microscale, so the crack branching inside the RVE can be properly considered. Once the crack propagation path is obtained, another numerical method (X-FEM/X-BEM) can be employed to model the crack in the macroscale. Crack branching has already been considered in peridynamics in [106]. A comparison against experimental results of X-FEM, cohesive models and peridynamics in dynamic fracture is done in [5], where it is observed that the peridynamics model is able to capture the physical behaviour seen in experiments.

A stochastic approach is likely to be the most useful way to model the extended fracture network, since the natural variability in geological conditions makes us unlikely to be able to obtain a deterministic model of the fracture system induced around any particular well. Moreover, the crack propagation obtained with the peridynamics formulation may change significantly if changes to the grid spacing or horizon size are made.
